# Substitution Effects in Aryl Halides and Amides into the Reaction Mechanism of Ullmann-Type Coupling Reactions

**DOI:** 10.3390/molecules29081770

**Published:** 2024-04-13

**Authors:** Rocío Durán, César Barrales-Martínez, Fabián Santana-Romo, Diego F. Rodríguez, Flavia C. Zacconi, Barbara Herrera

**Affiliations:** 1 Instituto de Investigación Interdisciplinaria (I3), Vicerrectoría Académica, Centro de Bioinformática, Simulación y Modelado (CBSM), Facultad de Ingeniería, Universidad de Talca, Campus Lircay, Talca 3460000, Chile; rocio.duran@utalca.cl (R.D.); cesar.barrales@utalca.cl (C.B.-M.); 2 Escuela de Química, Facultad de Química y de Farmacia, Pontificia Universidad Católica de Chile, Av. Vicuña Mackenna 4860, Macul, Santiago 7820436, Chile; fmsantana@espe.edu.ec (F.S.-R.); dfrodriguez1@uc.cl (D.F.R.); 3 Departamento de Ciencias Exactas, Universidad de las Fuerzas Armadas ESPE, Sangolquí 171103, Ecuador; 4 Institute for Biological and Medical Engineering, Schools of Engineering, Medicine and Biological Sciences, Pontificia Universidad Católica de Chile, Santiago 7820436, Chile; 5 Laboratorio de Química Teórica Computacional (QTC), Facultad de Química y de Farmacia, Pontificia Universidad Católica de Chile, Av. Vicuña Mackenna 4860, Macul, Santiago 7820436, Chile

**Keywords:** computational chemistry, conceptual DFT, reaction mechanisms, *N*-arylations, substituent effect

## Abstract

In this article, we present a comprehensive computational investigation into the reaction mechanism of *N*-arylation of substituted aryl halides through Ullmann-type coupling reactions. Our computational findings, obtained through DFT ωB97X-D/6-311G(d,p) and ωB97X-D/LanL2DZ calculations, reveal a direct relation between the previously reported experimental reaction yields and the activation energy of haloarene activation, which constitutes the rate-limiting step in the overall coupling process. A detailed analysis of the reaction mechanism employing the Activation Strain Model indicates that the strain in the substituted iodoanilines is the primary contributor to the energy barrier, representing an average of 80% of the total strain energy. Additional analysis based on conceptual Density Functional Theory (DFT) suggests that the nucleophilicity of the nitrogen in the lactam is directly linked to the activation energies. These results provide valuable insights into the factors influencing energetic barriers and, consequently, reaction yields. These insights enable the rational modification of reactants to optimize the *N*-arylation process.

## 1. Introduction

In 1901, Fritz Ullmann reported the first transition metal-mediated coupling reaction for C(sp^2^)-C(sp^2^) bond formation [1]. The scope of this reaction, catalyzed initially by stoichiometric amounts of copper, was extended by Irma Goldberg in 1906 for the *N*-arylation of amides [2]. However, these historical coupling reactions are usually carried out under rather harsh conditions, limiting their application [3]. Thus, the truly catalytic Ullmann reaction, called Ullmann-type, was only achieved until the end of the last century through the independent work of different research groups through the introduction of chelating N- and O-based bidentate ligands for Cu(I) in combination with weak inorganic bases [4,5,6]. 

The versatility and reliability of the Ullmann-type reaction for the formation of carbon–carbon and carbon–heteroatom bonds (C-N, C-O, C-S, and C-P) have made it an indispensable method in the pharmaceutical and agrochemical industries. Furthermore, it has been successfully implemented in the synthesis and functionalization of materials [7]. Unquestionably, one of the most important advantages over other methodologies for C-N couplings, such as palladium-based Buchwald–Hartwig Cross-Coupling Reaction, is the abundance and low toxicity of copper. Thus, the growing demand for sustainable and safe processes has driven demand for highly efficient catalytic reactions based on abundant first-row transition metals with a low environmental impact [8,9,10]. Although the Ullmann-type reaction has contributed to the development of sustainable processes, critical research areas seek to improve its efficiency and sustainability. For example, through highly active and reusable catalysts, second-generation ligands, energy-efficient activation methods, and the replacement of toxic and contaminating solvents [11,12].

**Scheme 1:**
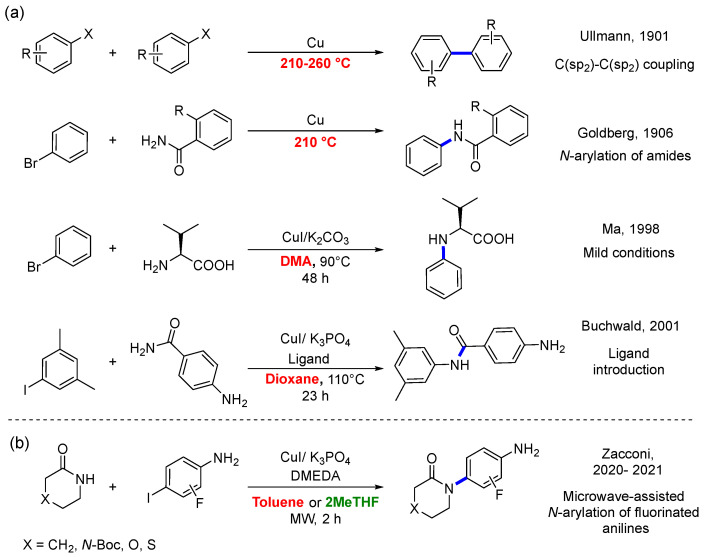
(**a**) Examples of breakthrough advances in the development of Ullmann-type coupling reactions. (**b**) Microwave-assisted synthesis of the Ullmann-type *N*-arylation using substituted aryl halides (2-fluoro-4-iodoaniline and 3-fluoro-4-iodoaniline), with different substituted lactams (X: *N*-Boc, O, S) and unsubstituted (X: CH_2_). In red: non-green solvents or high temperatures. In green: green solvents [1,2,4,5,13].

In this sense, Zacconi et al. reported a microwave-assisted synthesis of *N*-arylamides from the Ullmann-type reaction between substituted lactams and 2-fluoro-4-iodoaniline, as shown in Scheme 1b [13]. Specifically, they found that at room temperature, in toluene for 96 h, the reaction yields for lactams substituted with S, O, and *N*-Boc were found to be 10%, 15%, and 13%, while for the unsubstituted (CH_2_ group) lactam, it was negligible. Nevertheless, at 160 °C for 2 h, in a microwave-assisted process, the reaction yields reach their maximum values of 62%, 86%, 94%, and 90%, respectively. Specifically, the lactam substituted with oxygen exhibits the highest yield, leaving N-Boc with an intermediate yield, contrary to what classic chemistry would predict. (with the exclusion of the S system from the comparison due to differences in the experimental temperature employed) [13]. Encouraged by the previous results, the Zacconi group turned their attention to replacing toxic toluene. A solvent screening determined that 2-methyl-tetrahydrofuran (2-MeTHF) allows for the synthesis of fluorinated *N*-arylamides and access to a valuable set of building blocks in medicinal chemistry [13]. In this case, the identical group of substituted lactams, as depicted in Scheme 1b, was employed in conjunction with 3-fluoro-4-iodoaniline as the aryl halide, resulting in reaction yields of 68%, 61%, 90%, and 85% for It is noteworthy that, despite variations in both solvents (toluene and 2-MeTHF) and aryl halides (2-fluoro-iodoaniline and 3-fluoro-iodoaniline) between the two experimental investigations, consistent trends are observed regarding reaction yields. Specifically, the lactam substituted with oxygen exhibits the highest yield, while the unsubstituted lactam demonstrates the lowest yield (with the exclusion of the S system from the comparison due to differences in the experimental temperature employed).

**Scheme 2:**
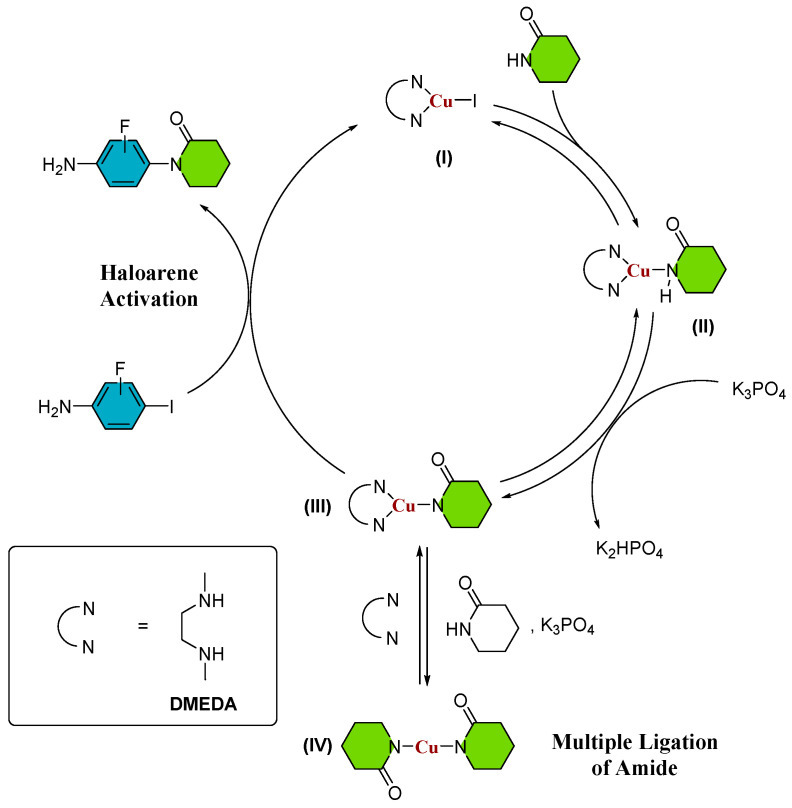
Ullmann–Goldberg catalytic cycle. The possible intermediates species are (I) Cu(I)Ln; (II) LnCu(I)-amide; (III) LnCu(I)-amidate (IV) Cu(I)-diamidate; Ln = DMEDA.

Furthermore, the authors have observed that substituting toluene with 2-MeTHF as the solvent in the reaction with 3-fluoro-iodoaniline leads to a significant enhancement in reaction yields. This observation underscores the potential of employing environmentally friendly solvents in conjunction with microwave heating as a promising alternative to traditional, hazardous solvents, resulting in a marked increase in reaction yields. However, it should be noted that while these findings have delineated conditions for optimizing reaction yields, the reactivity trends that impact reaction yields for various substituted lactams remain unclear. Consequently, a more comprehensive and rigorous analysis is imperative to attain a profound understanding of the underlying mechanisms and thereby facilitate informed and systematic enhancements in the process. Given the existence of multiple potential mechanisms for the Ullmann–Goldberg reaction [14,15,16,17,18,19,20,21,22,23], our study primarily focuses on investigating the catalytic cyclic pathway depicted in Scheme 2. The generally accepted mechanism for the Ullmann–Goldberg coupling reaction suggests that the CuI complex initially coordinates with the diamine ligand DMEDA (refer to Scheme 2, (I)). Subsequently, amide coordination leads to the formation of an intermediate (II), where the removal of protons from the N-H bond of the lactam is facilitated by an inorganic base (K_3_PO_4_ was employed in references [7,8,9,10,11,12,13]), resulting in the formation of a copper-amidate complex (III) [13,15]. The use of inorganic bases with limited solubility in conventional organic solvents enables the capture of generated protons during the reaction, thereby minimizing the formation of by-products. Eventually, intermediate (III) reacts with the aryl halide, leading to the desired *N*-arylated amide [24]. Although the mechanism might proceed in the absence of ligand [24,25], we present the use of diamine ligand, which promotes the formation of an activated copper (III) catalyst (Scheme 2) and becomes the aryl halide activation rate-limiting step as previously studied by our group [26], where it was found that the mechanism of C-N bond formation proceeds by a nucleophilic substitution in a single kinetic step. 

In the present work, we have studied the Ullmann coupling reaction considering four substituted lactams (X = CH_2_, S, O, and *N*-Boc), analyzed experimentally by Zacconi et al. [7,13], the experimental Cu-DMEDA catalyst, and two different substituted anilines, 2-fluoro-4-iodoaniline and 3-fluoro-4-iodoaniline, which we will label as **2F** and **3F**, respectively, resulting in eight different reactions. We analyzed the X substitution on the lactam and the fluorine position of the aniline molecule with the aim of determining the most important factors that govern the process that could be related to differences in reaction yields that the group found experimentally, fundamentally the differences in yield that N-Boc lactam had with **2F** and **3F**. To gain a thorough understanding of this reaction, we approached it from various theoretical angles: (i) using the Activation Strain Model of reactivity (ASM) [27,28] to examine the reaction mechanism; (ii) identifying interactions among reactants at the transition state using the non-covalent interactions (NCI) [29,30,31] index; and (iii) conducting a reactivity analysis using conceptual Density Functional Theory (cDFT) reactivity indexes [32,33,34].

### 1.1. Theoretical Background

#### 1.1.1. Conceptual Density Functional Theory (cDFT)

Conceptual DFT (cDFT) [32,33,34,35,36] is an area defined within the Density Functional Theory (DFT) [34], which provides a variety of response functions of the number of particles and the external potential, such as electronic chemical potential, chemical hardness, electrophilicity, and the Fukui function. These indexes are handy for obtaining insights into the intrinsic global and local reactivity of a molecular system, and they can also be computed along a reaction path to understand the changes in different molecular properties in reacting systems [32,33,35,36].

The electronic chemical potential (*μ*) [32,33] is a global reactivity index that describes the escaping tendency of electrons from an equilibrium electronic cloud, flowing the electrons from systems with a high chemical potential to a low chemical potential, in analogy to thermodynamics. It is defined as the derivative of the energy with respect to the number of electrons at constant external potential, such as [32,37]:

(1) $$\mu ={\left(\frac{\partial E}{\partial N}\right)}_{v\left(r\right)}$$

Equation (4) shows the formal definition of *μ.* Since *N* is a discrete variable, it must need approximations. The analytical expression to calculate the chemical potential comes from applying the finite difference approximation and the Koopmans theorem, as Equation (2) shows [37]:

(2) $$\mu \approx -\frac{I+A}{2}\approx \frac{\varepsilon_{LUMO}+\varepsilon_{HOMO}}{2}$$

Thus, it is possible to quantify the values of chemical potential from the energies of frontier molecular orbitals.

The chemical hardness (*η*) [33,36,37,38,39], is a global reactivity index that is associated with the ability of the system to resist the redistribution of its electron density. *η* can be associated with the stability of the system, being those systems that are less reactive in comparison to those systems with lower values of hardness. It is defined as the second derivative of the energy with respect to the number of electrons at constant external potential [33,37,38,39]:

(3) $$\eta ={\left(\frac{\partial^{2}E}{{\partial N}^{2}}\right)}_{v(r)}={\left(\frac{\partial \mu }{\partial N}\right)}_{v(r)}$$

Analogously to *μ*, the analytic expression for chemical hardness comes from the application of the finite difference approximation and the Koopmans theorem [37,38,39]:

(4) $$\eta \approx -\frac{I-A}{2}\approx \frac{\varepsilon_{LUMO}-\varepsilon_{HOMO}}{2}$$

Electrophilicity (*ω*) has been defined within conceptual DFT as a global index that measures the energetic stabilization of a chemical system when it is saturated with electrons from an external source [40]. Electrophilicity is expressed as:

(5) $$\omega =\frac{\mu^{2}}{2\eta }$$

where *μ* and *η* values can be extracted from Equations (2) and (4) [37]. Local electrophilicity, as its name indicates, is the local tendency to react towards electrons, and it can be condensed to the atom *k* (*ω_k_*) [41,42] by using the electrophilic Fukui function as:

(6) $$\omega_{k}=\frac{\mu^{2}}{2\eta }\;f_{k}^{+}=\omega f_{k}^{+}$$

This last Equation implies that the most electrophilic site is also the less hard (softer) site [41]. Finally, the multiphilic index is defined from the index of generalized Phylicity (*ω_k_^α^*) defined by Chattaraj et. al. and the dual descriptor (∆f) defined by Morell and Toro-Labbé as [42,43]:

(7) $$∆\omega_{k}=\left[\omega_{k}^{+}-\omega_{k}^{-}\right]=\omega ∆f_{k}$$

where $∆f_{k}$ is the condensed dual descriptor and ω is the electrophilicity index. The site is electrophilic when $∆\omega_{k}$ > 0, whereas $∆\omega_{k}$< 0, the site is nucleophilic. To calculate the local nucleophilicity and philicity condensed to atoms, we use the corresponding condensed fukui functions using the Yang and Mortier approximations [37,41,42].

#### 1.1.2. Activation Strain Model (ASM)

The activation strain model (ASM) is a reactivity model based on electronic density fragmentation, and it describes the physical nature of the activation energies observed in chemical reactions [27,28]. This formulation states that the total energy of the system is expressed as two contributions: the strain energy (${∆E}_{strain}$) and the interaction energy (${∆E}_{int}$), such as:

(8) $$∆E\left(\xi \right)={∆E}_{strain}\left(\xi \right)+{∆E}_{int}\left(\xi \right)$$

${∆E}_{strain}$ measures the energy associated with the geometrical distortion of each reactant separately, while ${∆E}_{int}$ represents the energy associated with the interaction between the distorted reactants.

#### 1.1.3. Non-Covalent Interactions Index (NCI)

The non-covalent interactions index (NCI) is a visualization tool that is based on the electron density (ρ(r)) and the reduced density gradient (RDG, s(r)), which corresponds to the first derivative of the electron density, and it is defined as [29,30,31]:

(9) $$s(r)=\frac{1}{2{\left(3\pi^{2}\right)}^{1/3}}\;\frac{\left|\nabla \rho (r)\right|}{{\rho (r)}^{4/3}}$$

The location of low-density zones and low gradients allows the identification of weak interactions in a molecular system, and higher densities lead to stronger interactions. To distinguish the stronger interactions, such as hydrogen bonds and steric interactions, it is necessary to consider the second derivatives of the density [29,30,31]. The sign of the second eigenvalue of the Hessian matrix ($\lambda_{2}$) indicates the strength of the interactions, where attractive interactions, such as hydrogen bonds, are identified with a negative $\lambda_{2}$, while repulsive steric interactions are represented by a positive number of $\lambda_{2}$ at interatomic regions [29,30,31].

## 2. Results

### 2.1. Energies and Reaction Profiles

**Figure 1:**
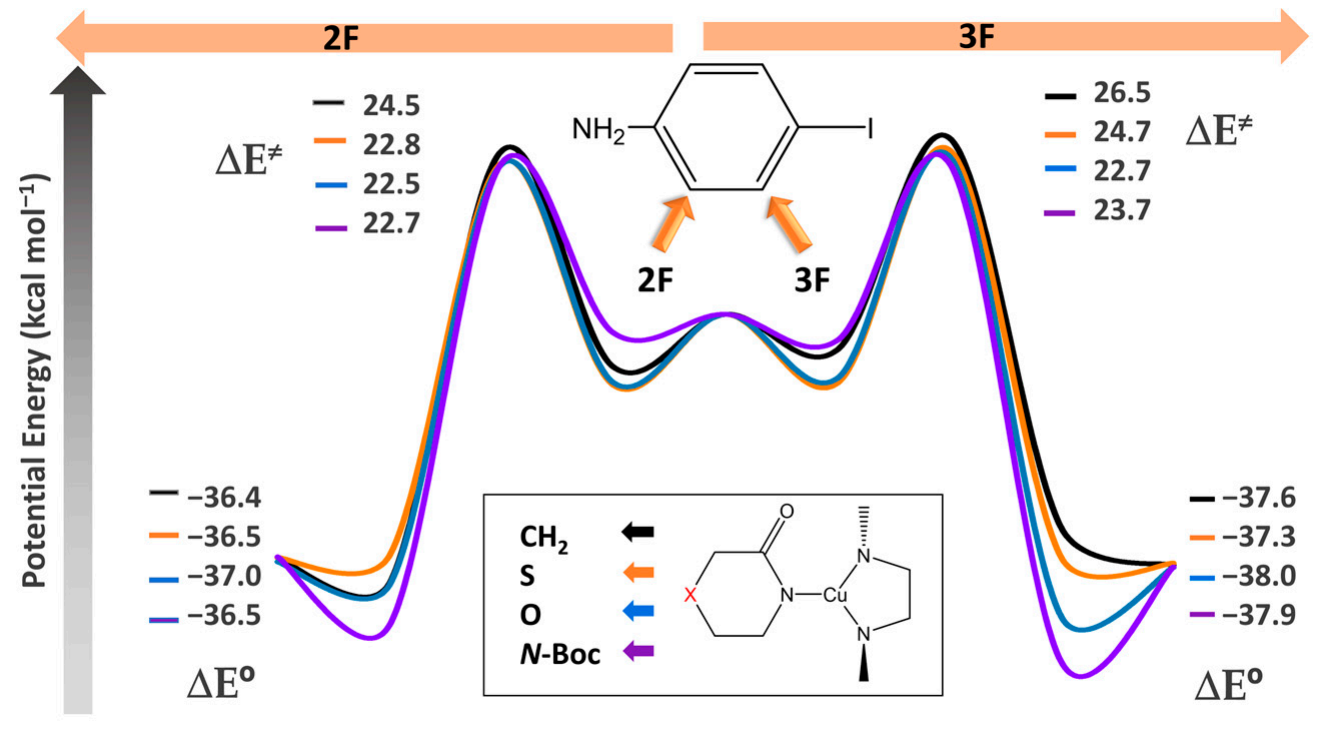
Energy diagram for the substitution of both the aryl halide in **2F** (left arrow) and **3F** (right arrow) and the substitution of the lactam complex with X: CH_2_, S, O, and *N*–Boc in each aniline substituted. All electronic energies are in kcal/mol.

In the context of the analysis of the Ullmann–Goldberg catalytic cycle, described in Scheme 2, we assumed that the reaction proceeds solely through coupling reactions without the occurrence of side reactions, following the mechanism obtained in reference [26]. In this sense, we have analyzed the rate determining step, which entails the coupling of the Cu-lactam complex with the fluorinated aniline and the subsequent recovery of the catalyst (called haloarene activation in Scheme 2). The reaction energies in the gas phase were determined for each X group in the lactam with **2F** and **3F** substituted anilines (indicated by left and right arrows in Figure 1, respectively). Our findings revealed that by keeping constant the X group in the lactam-Cu complex, the activation energy is lower in **2F** compared to **3F** for all the X groups in the lactam, with ∆∆E^≠^ of 2.0, 1.9, 0.2, and 1.0 kcal/mol for X = CH_2_, S, O, and N-Boc, respectively. Consequently, the activation energy exhibits an increasing order of O < N-Boc < S < CH_2_ for both F–iodoanilines, with values ranging from 22.5 to 24.5 kcal/mol in the case of **2F** aniline and from 22.7 to 26.5 kcal/mol in **3F** aniline.

**Figure 2:**
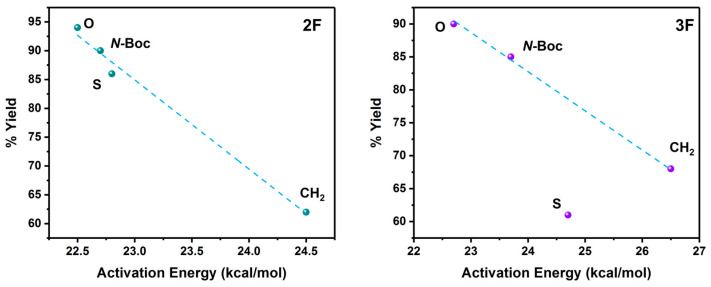
Reaction Yields versus activation energy for the reactions with **2F** (**left**) and **3F** (**right**) anilines. The reaction yield values were extracted from previous experimental works [7,13]. The dashed line in the relationship for **3F** is the linear fitting without including the S system.

Based on these reaction barrier values and the experimental reaction yields that were previously published by the Zacconi group [13,15], we have found a direct relationship between both quantities, as can be seen in Figure 2.

**Figure 3:**
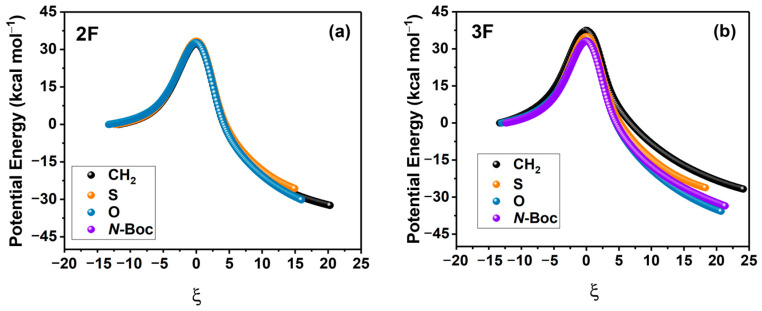
Potential energy profiles for **2F** (**a**) and **3F** (**b**) anilines. All values are in kcal/mol. IRC coordinates are measured in amu^1/2^ Bohr.

To gain insights into the nature of the activation energies and consequently the reaction yields associated with substitutions in both the aniline and Cu-lactam systems, we have analyzed in detail the potential energy profile of each reaction by decomposing the potential energy along the reaction coordinate using the ASM. To carry out this analysis, we first obtained the potential energy profiles for each coupling reaction with substitution in X (CH_2_, S, O, N-Boc), which are presented in Figure 3a,b for **2F** and **3F**, respectively. 

### 2.2. EDA and Strain Energy Analysis

**Figure 4:**
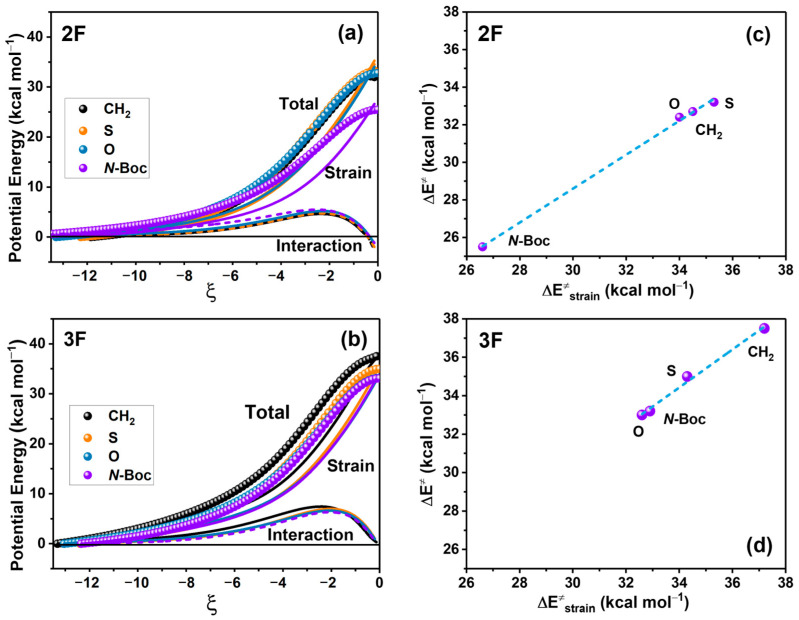
Energy profiles associated with the decomposition of energy related to strain (lines) and interaction (dots) for **2F** (**a**) and **3F** (**b**) in kcal/mol; IRC values are in amu^1/2^ Bohr. Activation energy vs. the strain energy of **2F** (**c**) and **3F** (**d**). $∆E^{\neq }=0.09{∆E}_{strain-TS}+1.51$; R^2^ = 0.99; and $∆E^{\neq }=0.98{∆E}_{strain-TS}+1.25$; R^2^ = 0.99, for **2F** and **3F**, respectively).

We conducted an energy decomposition analysis employing the ASM approach, as illustrated in Figure 4 for **2F** and **3F** anilines. The aim of this analysis was to identify the contribution of the strain and interaction energies to the reaction energies in Figure 4a and the strain contribution to the reaction barrier in Figure 4b.

**Figure 5:**
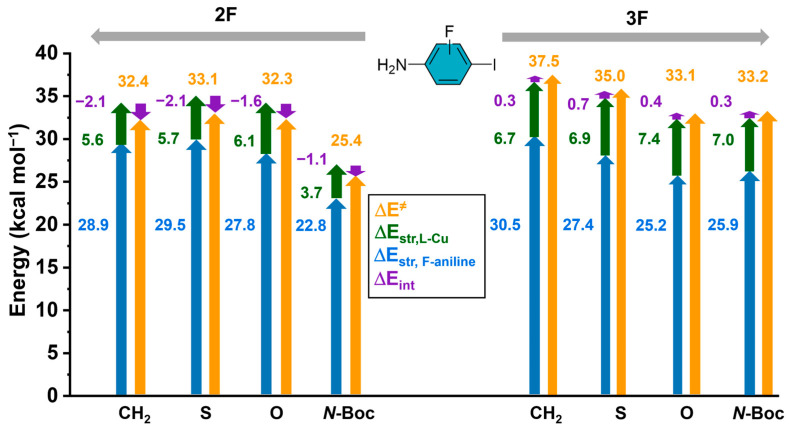
Activation energy $\left(∆E^{\neq }\right)$ yellow arrow, strain energy of halogenated aniline (${∆E}_{\begin{array}{c} strain-F-aniline \end{array}}$) blue arrow, strain energy of the substituted lactam-Cu complex (${∆E}_{strain-L-Cu}$) green arrow, interaction energy (${∆E}_{int}$) purple arrow. All strain energies were calculated at the transition state structure. All energy values are in kcal/mol.

To gain further insights into the nature of this strain energy, we partitioned the contributions, considering the transition state complex treated as an ensemble of two reacting fragments, the aniline (**2F** or **3F**) and the Cu-lactam. The results of the overall contributions to the barriers are presented in Figure 5, and it can be seen that the largest contribution to the strain energy comes from the iodo-anilines (**2F** and **3F**) in contrast to the Cu-lactam species. The interaction energies are almost negligible; however, they only present negative or stabilizing values in **2F.**

**Figure 6:**
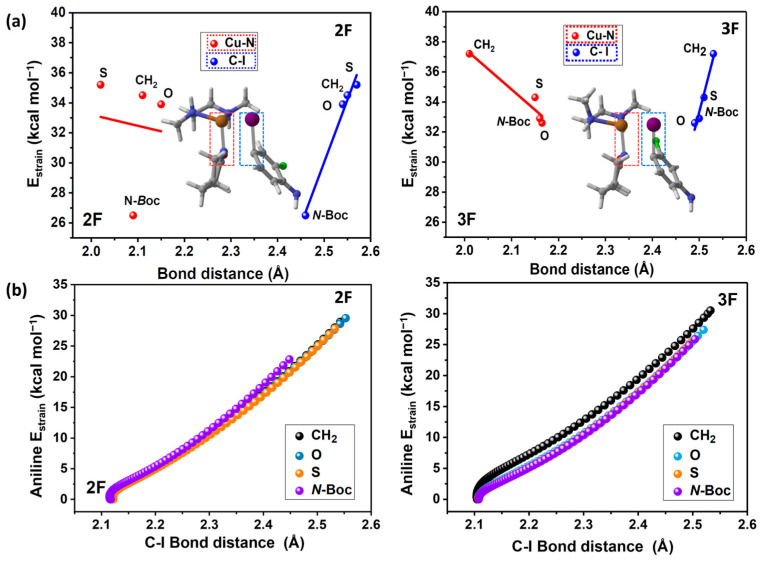
(**a**) Strain energy (strain aniline + strain Cu-lactam complex) vs. Cu-N (Cu-lactam complex) and C-I (halogenated aniline) internuclear distances $∆E_{strain}=120d_{C-I,TS}-268$; R^2^ = 0.96 $∆E_{strain}=-27.1d_{Cu-N,TS}+91.8$; R^2^ = 0.92 for **2F** (without considering *N*-Boc) and $∆E_{strain}=83.1d_{C-I,TS}-177$; R^2^ = 0.98; $∆E_{strain}=-9.62d_{Cu-N,TS}+54.7$; R^2^ = 0.97, for **3F**. (**b**) Halogenated iodoaniline strain energy vs. C-I bond distance at each point of the reaction coordinate.

To keep analyzing the strain energies, we have analyzed the transition state complexes considering the two fragments, the iodoaniline (**2F** and **3F**), and the Cu-L. To achieve this task, we plotted the Cu-N and the C-I distances at the transition state versus their strain energies in Figure 6a. Figure 6b depicts the aniline strain energy from the reactants to the TS versus the evolution of the C-I bond along the reaction path.

### 2.3. NCI Analysis

**Figure 7:**
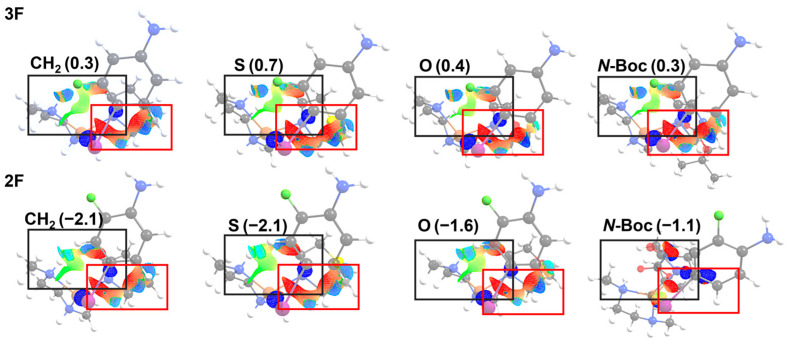
NCI 3D for the transition state structures of the X: CH_2_, S, O, and N-Boc were obtained in the reactions with substitution in both **3F** and **2F** of the aryl halide: Red: steric interactions; green: van der Waals interactions; blue: strong ionic interactions. The value in parenthesis corresponds to the interaction energies obtained by using ASM (kcal mol^−1^).

In conjunction with strain energy analysis, recognizing its role as the primary determinant of the reaction barriers, we have conducted a comprehensive 3D NCI index analysis on all transition state structures, as illustrated in Figure 7. 

The Figure 7 shows the TS, where the complex lies perpendicular to the iodoaniline and the lactam planes in all the **2F** and **3F** systems. The black rectangle indicates the location of stabilizing van der Waals (green) and ionic (blue) interactions, located mostly between the Cu complex and the lactam. The red rectangle is delimiting the repulsion interactions, mainly located between the lactam and iodoaniline rings in all reacting systems. Note that the 2F with N-Boc substitution does not present a large repulsion interaction, according to the lower values of strain energies at the TS.

### 2.4. Local Electrophilicity

To provide a comprehensive understanding of the interacting sites in the transition states, we have performed a complementary analysis involving reactivity indexes using c-DFT (refer to Table S1 in the supporting information). Specifically, we examined the multiphilicity index (${∆\omega }_{N}$) of the nitrogen atom at the lactam-copper complex (or the nucleophilic site) and the local electrophilicity ($\omega_{C}$) of the reacting carbon atom in the F-iodoaniline.

It is interesting to note that the electrophilicity of the carbon atom does not play an important role in defining the reaction barrier in the **2F** system. In contrast, in **3F,** the fluorine substitution lowers the barriers along with the electrophilicity of C, showing that the fluorine substitution in meta plays a synergic role with the amino group activating the electrophilicity of the carbon atom, especially in *N*-Boc. 

## 3. Discussion

### 3.1. Energies and Reaction Profiles

When analyzing the lactam-copper reactants in Figure 1, the activation energies were determined to be 22.5 and 22.7 kcal/mol with the **2F** and **3F** anilines, respectively. The lowest barrier is presented at the oxygen substitution of the Cu-lactam reactant complex, kinetically favoring both reactions. Conversely, the least favorable reaction corresponds to the unsubstituted Cu-lactam complex, with activation energies of 24.5 and 26.5 kcal/mol for the **2F** and **3F** anilines, respectively. 

Based on the calculated reaction barrier values and the experimental reaction yields [7,14] we have found a direct relationship between both quantities, as can be seen in Figure 2. These findings emphasize the impact of substituent modifications in the Cu-lactam complex and the use of halogenated anilines. 

We also plotted the potential energy profiles for each coupling (Figure 3a,b). It is observed that all reactions present a similar behavior along the IRC, which is an indicator of a reaction consisting only of an elemental step, and there are no further differences along the substitutions other than the reaction barriers. Indicating that the structural changes in the reactive systems induce variations in the reactivity of the chemical species, thus affecting the activation energies in a chemical transformation and directly influencing the resulting reaction yields. In order to further investigate the effects of either the lactam substitution or the fluorine position in the aniline, we will discuss the impact of those changes at the EDA and the strain energy along the reaction coordinate.

### 3.2. EDA and Strain Energy Analysis

We conducted an energy decomposition analysis employing the ASM approach, as illustrated in Figure 4 for **2F** and **3F** anilines. This analysis indicates that strain energy primarily dictates the potential energy profile of reactants in the transition state structure. Variations in strain energy are consistent with the changes at the energy barriers among the reactions. In contrast, the contribution of the interaction energy to the potential energy is notably lower and remains relatively consistent across all reactions.

A more detailed examination of this energetic decomposition at the transition state (TS) affirms the substantial role of strain energy in determining the activation energy for both **2F** and **3F** substitutions, revealing a linear correlation between these factors (refer to Figure 4b). In contrast, the contribution of interaction energy at TS is nearly negligible for **3F**, whereas **2F** exerts a minor stabilizing effect with values of −2.1, −2.1, −1.6, and −1.1 kcal/mol at CH_2_, S, O, and N-Boc substitutions, respectively. 

The remarkable linear correlation (R^2^ = 0.99 for both substituted anilines) observed between the strain energy at TS and the energy barrier suggests that an increase in strain energy resulting from lactam substitution leads to a corresponding rise in the activation energy of the process (see Figure 4b). It is worth noting that the activation energy values presented here may not precisely match those in the previous section due to the consideration of the initial reactant complex in this analysis.

To gain further insights into the nature of the $∆E_{strain}$, we decomposed it into the contributions of the iodoaniline (**2F**, **3F**) and the Cu-lactam fragments, along with comparing their contributions to the $∆E_{int}$ and $∆E^{\neq }$.

The results, presented in Figure 5, demonstrate that the distortion exhibited by the halogenated aniline contributes significantly to the overall strain energy (77–82% for **3F**, 82–86% for **2F**). These findings suggest that the ring and substitution pattern in the iodoaniline and, to some extent, the electronic effects from the lactam substituents influence mainly the deformation of the reactant complex (for further details, see Table S1). For instance, if we analyze the *N*-Boc substituted lactam-Cu complex, the presence of *N*-Boc induces a lower geometrical distortion of the aniline reaction partner compared to the unsubstituted (X: CH_2_) lactam-Cu complex, which implies a lower activation energy.

The primary structural changes associated with strain energy in both fragments involve the elongation of the Cu-N bond in the lactam-copper complex and the C-I bond in the substituted aniline. The relationship between these geometric parameters and the total strain energy is displayed in Figure 6a, where a direct linear correlation is only observed in the case of the C-I bond of the aniline (blue line), while the elongation of the Cu-N bond exhibits an inverse correlation with the strain energy in almost all cases (red line), confirming that the aniline strain is the factor that governs the total strain energy and the activation barriers. This direct relationship between strain energy and the C-I internuclear distance of the aniline persists throughout the entire reaction pathway, as depicted in Figure 6b, where a linear correlation is observed between these two quantities from the initial stage of the reaction to the transition state. This indicates that an elongation of the C-I bond augments the strain energy and, consequently, the activation energy.

### 3.3. NCI Analysis

On the other hand, even though interaction energy has a lower contribution to the activation energies, it is important to investigate the variations in the interaction energy among the halogenated anilines, keeping the X group constant (refer to Figure 5). To accomplish this, we performed a non-covalent interactions analysis employing the NCI index in all the transition state systems, as depicted in Figure 7. Our analysis reveals that weak attractive van der Waals interactions (green surface in the black box) in the TS structures exhibit greater strength at the **2F** for reactions with X = CH_2_, S, and O, suggesting a dominant role of attractive interactions in the overall interaction energy. In the case of N-Boc, the interaction energy is attributed to a reduction in repulsive interactions (indicated by the red color in the red box) compared to N-Boc at the **3F**, affecting the strain energies of the aniline already discussed in Section 3.2. These findings suggest that in reactions involving **2F** anilines, the interaction energy has a stabilizing effect, whereas in reactions with **3F** anilines, it has a destabilizing influence, in agreement with the $∆E_{strain}$ results. This difference in interaction energy contributes to the preference for reactions with **2F** substitutions in terms of kinetic favorability over those with **3F** substitutions.

### 3.4. Local Electrophilicity

To complement electronic information on the substituent effects on both the Cu-lactam complex, we examined the multiphilicity index (${∆\omega }_{N}$) at the nucleophilic nitrogen and the local electrophilicity in the carbon atom bonded to the leaving group in the **2F** and **3F** iodoanilines ($\omega_{C}$).

**Figure 8:**
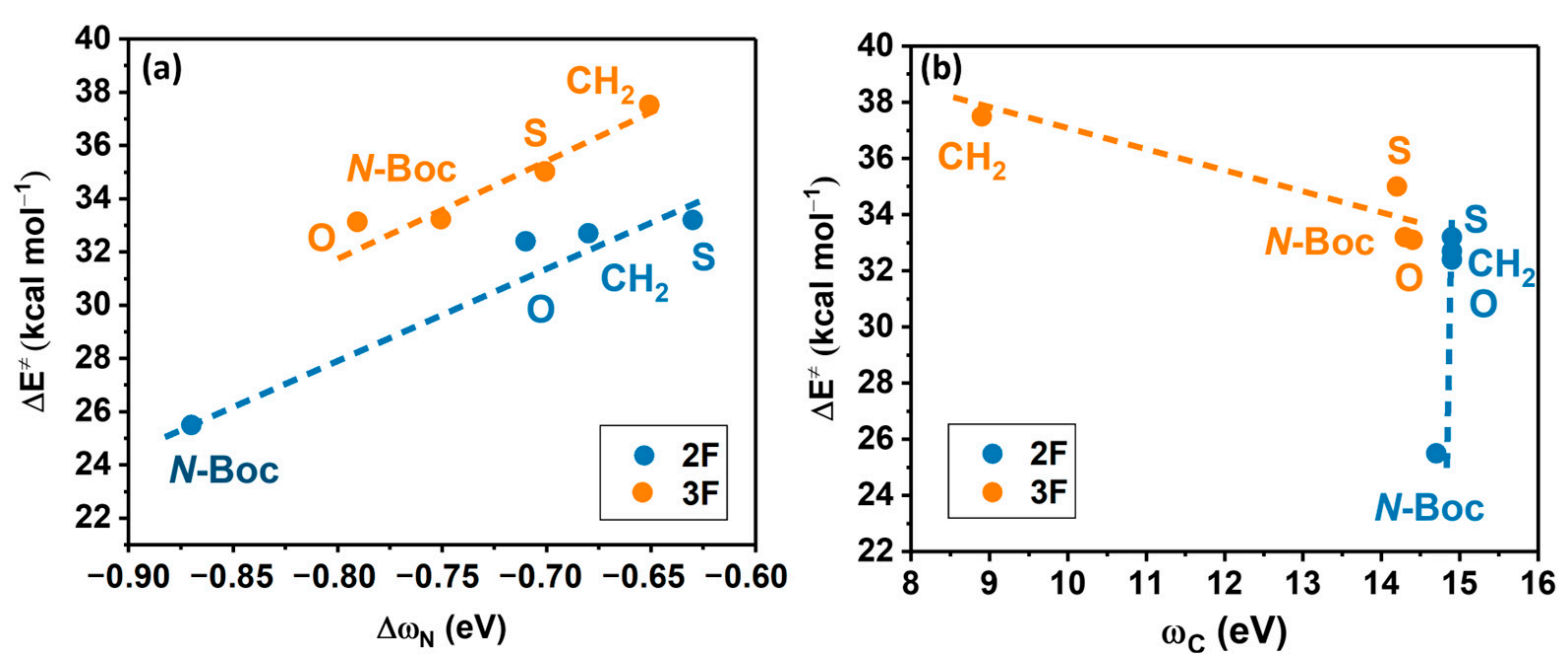
(**a**) Activation energy (∆E^≠^) vs. condensed-to-nitrogen multiphilicity. **2F**: ${∆E}^{\neq }=34.2{∆\omega }_{N}+55.6$; R^2^ = 0.95 and **3F:** ${∆E}^{\neq }=32.4{∆\omega }_{C}+58.1$; R^2^ = 0.91, (**b**) Activation energy vs. condensed-to-carbon electrophilicity; **3F**: ${∆E}^{\neq }=-0.7{∆\omega }_{C}+43.8$; R^2^ = 0.84.

The primary results obtained for the substitution of the halogenated aniline in the **2F** and **3F** systems indicate a direct linear correlation of |${∆\omega }_{N}$| versus ${∆E}^{\neq }$, as illustrated in Figure 8a. Regarding $\omega_{C}$ and its relationship to ${∆E}^{\neq }$ as shown in Figure 8b, we observe only an inverse linear correlation at **3F**. It is important to note a significant deviation in the local electrophilicity values between the unsubstituted lactam complex and the substituted systems, namely 8.9 vs. 14.2–14.3 eV, respectively.

For the **2F** system, $\omega_{C}$ remains quite constant across all four systems, implying that differences in activation energy are not attributed to changes in the reactivity of the electrophilic site. Indicating that the reaction is only directed by the lactam substitution, affecting directly the nucleophilicity of the nitrogen. The electronic influence of the fluorine substitution is only marginal, considering this atom is a weak o-deactivating of the carbon atom in the **3F** molecule and plays no role in the carbon electrophilicity in **2F**.

**Figure 9:**
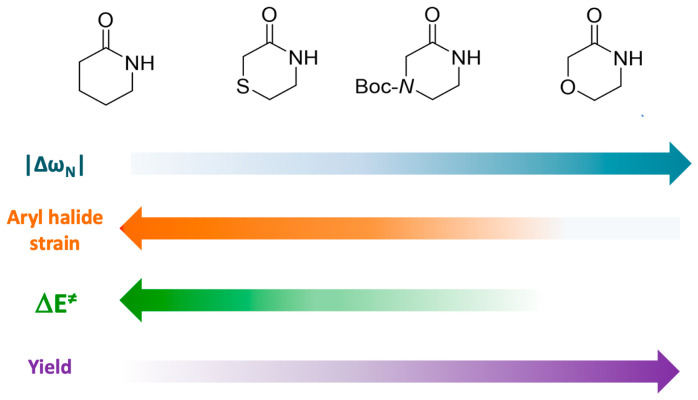
Reactivity scale that relates the nucleophilicity of nitrogen in lactams, strain of aryl halide, and activation energy with the reaction yields of the studied Ullmann–Goldberg coupling reaction.

In summary, the observed differences in activation energies for coupling reactions, associated with various substitution patterns in halogenated aniline and the Cu-lactam complex, can be attributed to several factors. These factors include lower strain energy, increased stabilizing interactions at the TS, and the nucleophilic and electrophilic reactivity of the reacting atoms. The key factors affecting reaction yields are visualized in Figure 9, representing a reactivity scale based on four substituted lactams. 

## 4. Materials and Methods

### Computational Details

The calculations were conducted employing Density Functional Theory (DFT) [33] to account for the electronic correlation in molecular systems. Specifically, the GGA-hybrid functional with long-range corrections ωB97X-D47 was used. Standard basis set functions 6-311G(d,p) [43,44] were employed to handle small atoms, while for copper and iodine, and LANL2DZ [45] as a quasi-relativistic pseudopotential and basis set for Cu atoms. The choice of the ωB97X-D/6-311G(d,p) and ωB97X-D/LanL2DZ methods for the calculations is based on specific considerations regarding the properties and systems under investigation. Specifically, wB97X-D was chosen for its demonstrated capability to accurately describe interactions in a wide range of systems, such as molecular aggregates, hydrogen complexes, and π–π systems. 6-311G(d,p) basis set provides an adequate description of molecular electronic structure, while LanL2DZ enables more efficient calculations without significantly compromising precision for the electronic structure of molecules containing transition metals.

To ensure the reactants and products exist as minima on the potential energy surface and the transition state (TS) corresponds to a first-order saddle point with a single negative eigenvalue on its Hessian matrix [46], a vibrational harmonic analysis was performed. For an in-depth investigation of the Ullmann coupling reaction mechanism, the intrinsic reaction coordinate (IRC) method [47,48,49] was employed. The analysis of intermolecular interactions was conducted using the non-covalent interaction index (NCI) [29,30,31] employing the NCIPLOT 3.0 program [29]. All calculations were carried out using Gaussian 16 [50] and Orca 4.0. [51,52] software packages. All the electronic and energetic values along the IRC to calculate the cDFT indexes were obtained by single-point calculations using the Gaussian 16 [49] package. Molecular structures were visualized and generated using the Chemcraft program [53], and figures were generated using the CYLview 10.b software [54].

## 5. Conclusions

The experimental reaction yields in the *N*-arylation of aryl halides through an Ullmann-type coupling reaction exhibit a direct correlation with the activation energy of the haloarene activation step within the catalytic cycle. This suggests that improving reaction yields can be achieved by reducing activation barriers.

A detailed analysis of the reaction mechanism, involving the decomposition of activation energy, highlights the predominance of strain energy in all cases. This predominance is attributed to the greater distortion of the aryl halide compared to the lactam-copper complex at the transition state. In contrast, interaction energy contributes exclusively to the activation energy in the case of the **2F** substituted aryl halide, playing a stabilizing effect through weak attractive van der Waals interactions, which makes the barrier and yield trends change from **2F** to **3F** systems due to the stabilization given by the geometry of N-Boc in the first set of systems.

Furthermore, conceptual density functional theory (DFT) reactivity indexes were utilized to elucidate the observed variations in activation energy. For the **3F** halogenated aniline, the activation energy correlates with the nucleophilic and electrophilic properties of the reactive sites in the lactam-copper complex and aryl halide, respectively. Conversely, for the **2F**-halogenated aniline, the activation energy is solely associated with the nucleophilicity of the nitrogen in the lactam-copper complex.

In conclusion, a higher level of condensed-to-nitrogen multiphilicity corresponds to lower strain energy in the aryl halide, leading to decreased activation energy and consequently an improved reaction yield.

## Data Availability

The original data presented in the study are openly available in the Supplementary Materials.

## Supplementary Materials

The following supporting information can be downloaded at: https://www.mdpi.com/article/10.3390/molecules29081770/s1. Further computational details, Tables S1–S3, Figures S1–S26 and the structures of the studied systems [30,31,43,44,45,46,47,48,49,50,51,52,53,54,55,56].
