# Supplementary material for: Substitution Effects in Aryl Halides and Amides into the Reaction Mechanism of Ullmann-Type Coupling Reactions
Source: Molecules. 2024 Apr 13;29(8):1770. doi: 10.3390/molecules29081770 (PMC11051942; doi:10.3390/molecules29081770)
Supplement: Supplementary file 1 [file molecules-29-01770-s001.zip › molecules-2948513-supplementary.pdf]

Substitution Effects in Aryl Halides and Amides into the Reaction Mechanism of Ullmann-Type Coupling Reactions

Supporting Information

Rocío Durán [1], César Barrales-Martínez [1], Fabián Santana-Romo [2,3], Diego F. Rodríguez [2], Flavia C. Zacconi [2,4,*] and Barbara Herrera [5,*]

[1] Instituto de Investigación Interdisciplinaria (I3), Vicerrectoría Académica, Centro de Bioinformática, Simulación y Modelado (CBSM), Facultad de Ingeniería, Universidad de Talca, Campus Lircay, Talca 3460000, Chile; rocio.duran@utalca.cl (R.D.); cesar.barrales@utalca.cl (C.B.-M.)
[2] Escuela de Química, Facultad de Química y de Farmacia, Pontificia Universidad Católica de Chile, Av. Vicuña Mackenna 4860, Macul, Santiago 7820436, Chile; fmsantana@espe.edu.ec (F.S.-R.); dfrodriguez1@uc.cl (D.F.R.)
[3] Departamento de Ciencias Exactas, Universidad de las Fuerzas Armadas ESPE, Sangolquí 171103, Ecuador
[4] Institute for Biological and Medical Engineering, Schools of Engineering, Medicine and Biological Sciences, Pontificia Universidad Católica de Chile, Santiago 7820436, Chile
[5] Laboratorio de Química Teórica Computacional (QTC), Facultad de Química y de Farmacia, Pontificia Universidad Católica de Chile, Av. Vicuña Mackenna 4860, Macul, Santiago 7820436, Chile
* Correspondence: fzacconi@uc.cl (F.C.Z.); bherrera@uc.cl (B.H.)

Content

1. Computational details …………………………………………………………….…. S2

2. Additional computational results ……………….……………………………………... S3

3. Cartesian coordinates……………………………………... S7

1. Computational details

The calculations were conducted employing Density Functional Theory (DFT) [3] to account for the electronic correlation in molecular systems. Specifically, the GGA-hybrid functional with long-range corrections ωB97X-D[1] was used. Standard basis set functions 6-311G(d,p) [43,44] were employed to handle small atoms, while for copper and iodine, the LANL2DZ [45] was employed as a quasirelativistic pseudopotential and basis set. To ensure the reactants and products exist as minima on the potential energy surface, and the transition state (TS) corresponds to a first-order saddle point with a single negative eigenvalue on its Hessian matrix, [46] a vibrational harmonic analysis was performed. For an in-depth investigation of the Ullmann coupling reaction mechanism, the intrinsic reaction coordinate (IRC) method [47-49] was employed. The analysis of intermolecular interactions was conducted using the non-covalent interaction index (NCI) [29-31] employing the NCIPLOT 3.0 program [29,56] All calculations were carried out using Gaussian 16 [50] and Orca 4.0 [51,52] software packages. Molecular structures were visualized and generated using the Chemcraft program[53] and the figures were generated using the CYLview software[54].

The choice of the ωB97X-D/6-311G(d,p) and ωB97X-D/LanL2DZ methods for the calculations is based on specific considerations regarding the properties and systems under investigation. These methods were selected due to their abilities to adequately describe dispersion interactions, which are relevant to the system under study. The ωB97X-D [55] functional was chosen for its demonstrated capability to describe dispersion interactions accurately and efficiently across a wide range of molecular systems. This functional has been specifically designed to enhance the description of such interactions, rendering it suitable for non-covalent systems such as molecular aggregates, hydrogen complexes, and π-π systems.

Furthermore, the combination of ωB97X-D with the 6-311G(d,p) and LanL2DZ basis sets was chosen for its balance between precision and computational cost. The 6-311G(d,p) triple-ζ basis set is relatively large and provides an adequate description of molecular electronic structure, while LanL2DZ is a double-ζ basis set, although smaller, it enables more efficient calculations without significantly compromising precision for the electronic structure of molecules containing copper when compared with a triple-ζ all electron basis set.

Additionally, a comparative analysis was carried out to evaluate the efficacy of various DFT functionals, including a hybrid-GGA, B3LYP; a hybrid-meta-GGA, M06-2X; a GGA, PBE; and three long-range corrected hybrid-GGA, ωB97X-D and ωB97X-D3. These were juxtaposed with the domain-based local pair-natural orbital DLPNO-CCSD(T) method and the SCS-MP2 method implemented in the Orca 4.0.0.2 software with the basis cc-pVTZ. The findings, depicted in Table 1, demonstrate that the ωB97X-D functional exhibits closer alignment with the high-precision DLPNO-CCSD(T) and SCS-MP2 methods in terms of activation energy of the process X: CH_2 with 3F.

Table S1: Activation energy ($\Delta E^{\neq}$) for different functionals of the Jacob ladder according to our benchmark work for this article.

Method	$\Delta E^{\neq}$ (kcal/mol)
B3LYP	41.0
M06-2X	24.2
PBE	30.0
ωB97X-D	26.5
ωB97X-D3	32.3
SCS-MP2	28.0
DLPNO-CCSD(T)	25.6

2. Additional Computational Results

Table S2: Activation energy, strain energy of halogenated aniline, strain energy of the substituted lactam complex, total strain energy, local mutiphilicity, and local electrophilicity. All strain energies were calculated at the transition state structure. All energy values are in kcal mol^{-1}, while the Δw_N and w_C are in eV.

Aryl halide	X	$\Delta E^{\neq}$	ΔE_{strain} Aniline	ΔE_{strain} Lactam Complex	ΔE_{strain} Total	E_{Int}	$\Delta\omega_N$	ω_C
2F	CH_2	32.7	28.9 (84%)	5.6 (16%)	34.5	-2.1	-0.68	14.9
	S	33.2	29.5 (84%)	5.7 (16%)	35.3	-2.1	-0.63	14.9
	O	32.4	27.8 (82%)	6.1 (18%)	34.0	-1.6	-0.71	14.9
	N-Boc	25.5	22.8 (86%)	3.7 (14%)	26.6	-1.1	-0.87	14.7
3F	CH_2	37.5	30.5 (82%)	6.7 (18%)	37.2	0.3	-0.65	8.9
	S	35.0	27.4 (80%)	6.9 (20%)	34.3	0.7	-0.70	14.2
	O	33.1	25.2 (77%)	7.4 (23%)	32.6	0.4	-0.79	14.4
	N-Boc	33.2	25.9 (78%)	7.0 (22%)	32.9	0.3	-0.75	14.3

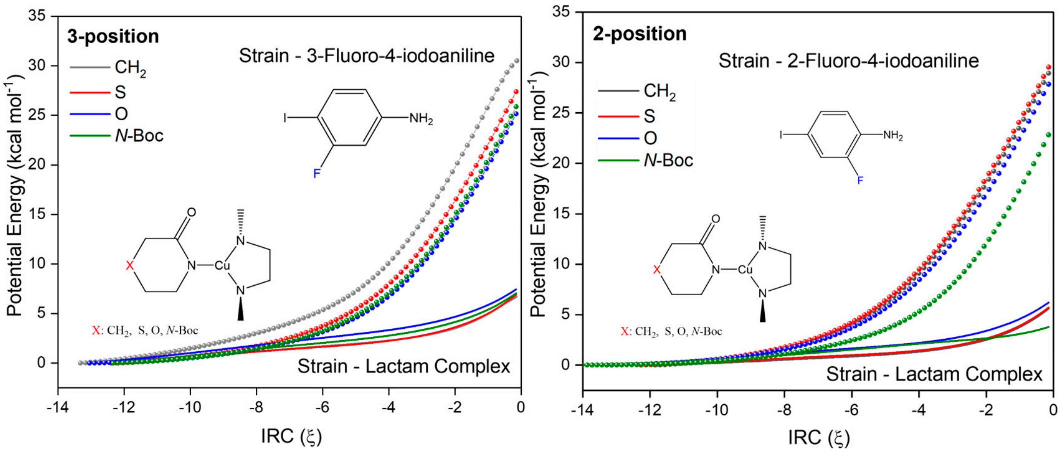

Figure S1: Strain energy profiles decomposed into contributions associated with the deformation of halogenated aniline (dots) and lactam complexes (lines).

Table S3: Experimental reaction yields of all reactions studied in this work. * K_3PO_4 (2.0 Eq.) was used as the base for all reactions.

Aryl halide	X	Methodology	Time (h)	Treatment Eq*	Yield (Toluene)	Yield (2-MeTHF)
**2F**	CH_2	MW	2	0.20 CuI, 0.40 DMEDA	17%	80%
	S	MW	2	0.20 CuI, 0.40 DMEDA	31%	80%
	O	MW	2	0.20 CuI, 0.40 DMEDA	61%	97%
	N-Boc	MW	2	0.20 CuI, 0.40 DMEDA	40%	90%
**3F**	CH_2	MW	2	0.20 CuI, 0.40 DMEDA	32%	48%
	S	MW	2	0.20 CuI, 0.40 DMEDA	56%	61%
	O	MW	2	0.20 CuI, 0.40 DMEDA	45%	80%
	N-Boc	MW	2	0.20 CuI, 0.40 DMEDA	46%	65%

	f^-	f^+	Δf
**CH_2**	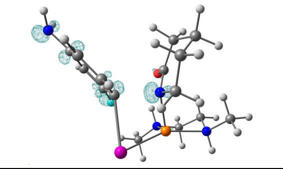	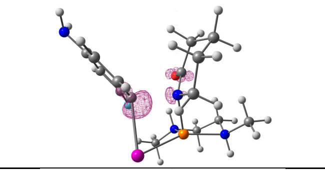	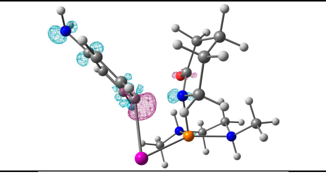
**O**	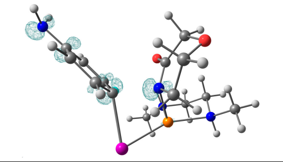	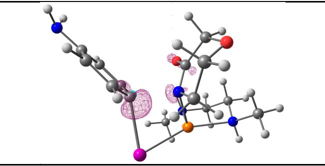	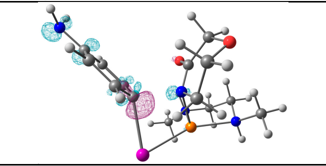

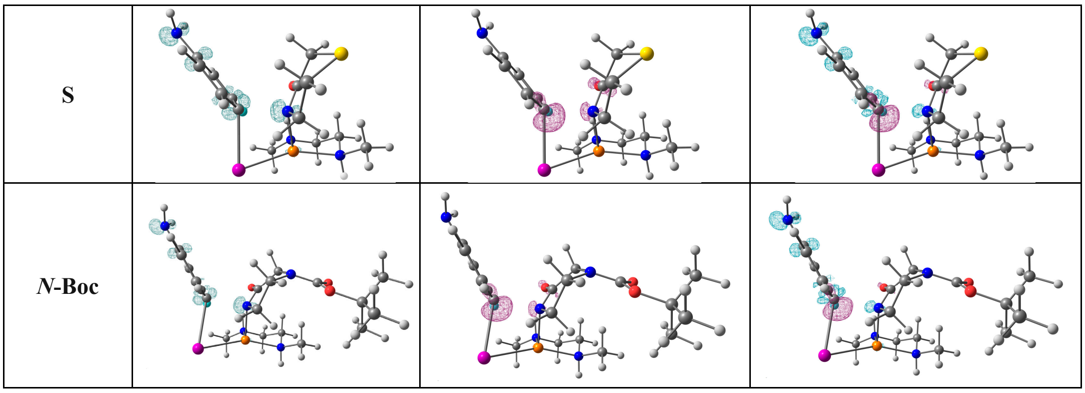

Figure S2: Fukui nucleophilic (f^-), electrophilic(f^+) and dual descriptor (Δf) at the TS in all studied systems.

The nucleophilicity of the nitrogen and the electrophilicity of carbon were computed at the transition state (TS) structure in each case. The effect of the *N*-Boc group in the calculation is mainly steric, resulting in a greater distortion of the complex and thus influencing indirectly the nucleophilicity of the reactive nitrogen atom at the TS. Electronically, the *N*-Boc group does not alter the frontier molecular orbitals, so the cDFT indices show low dependence on its presence. Figure S2 displays the Fukui functions and the dual descriptor, indicating that the *N*-Boc group does not alter in a large extent these reactivity descriptors, which are localized in the same reactive centers, regardless the X group.

Regarding the molecular orbitals and electrostatic surface at each substituted lactam, we plotted the HOMO, LUMO and ESP for all our systems in Figure S3. It can be noted that there are no significant differences were found among all the lactams and might not indicate the effect of the solvent in any system. It must be addressed however, these isolated reactants are not the reactive molecules, but rather the lactam-cooper complexes coming from the interaction of the lactam and the catalyst.

X	*HOMO*	*LUMO*	ESP
CH_2	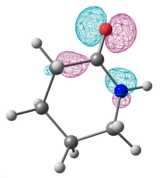	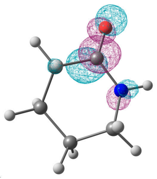	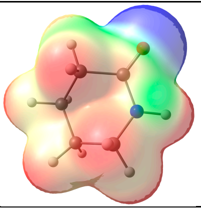
O	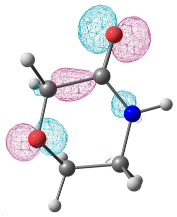	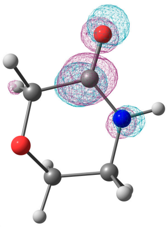	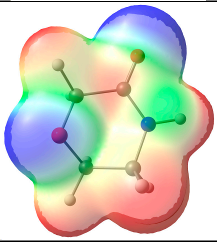
S	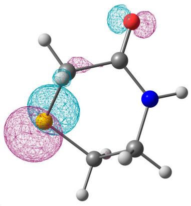	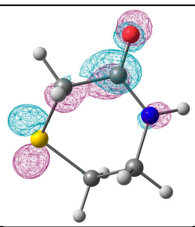	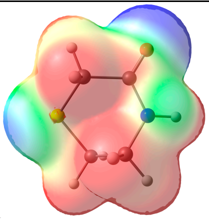
N-Boc	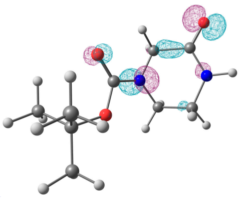	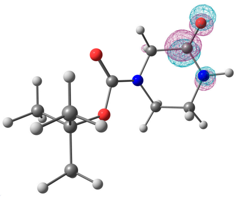	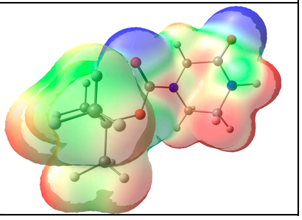

Figure S3: HOMO, LUMO and ESP isosurfaces of the studied lactams

3. Cartesian coordinates

Cartesian coordinates using wB97XD in combination with the 6-311G(d,p) basis set, and the LANL2DZ was used as a quasirelativistic pseudopotential and basis set. Optimized geometries are given below in standard XYZ format. The total number of atoms is shown before the cartesian coordinate in each case.

Isolated reactants – Copper complex lactam

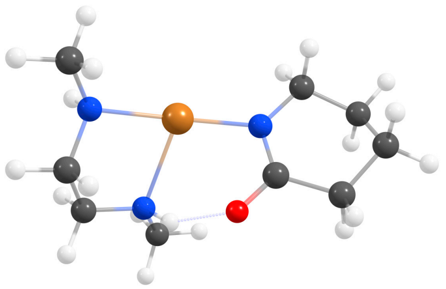

Figure S4: Lactam-copper complex unsubstituted (X: CH_2) computed structure in gas phase.

#p opt freq wB97XD/gen pseudo=read nosymm int=(grid=ultrafine)

34

Cu	0.920968000	0.218306000	2.381709000
N	2.652749000	-0.820053000	2.589142000
N	0.131499000	-2.008374000	1.975475000
C	-0.994665000	2.587102000	2.397278000
C	-0.823026000	1.010055000	0.540442000
C	-2.346010000	3.032246000	1.849960000
H	-1.077351000	2.391929000	3.471282000
C	-1.913566000	1.749052000	-0.228112000
C	-2.267606000	3.123146000	0.331873000
H	-3.116008000	2.303843000	2.132483000
H	-1.590817000	1.797413000	-1.269997000
N	-0.452309000	1.397988000	1.756051000
O	-0.321644000	0.005059000	-0.017550000
C	2.549843000	-1.957466000	1.650103000
H	2.465789000	-1.539845000	0.642547000
H	3.458728000	-2.573409000	1.686573000
C	1.329722000	-2.817326000	1.956260000
H	1.450554000	-3.279855000	2.945811000

H	1.295334000	-3.644271000	1.227458000
H	-2.793256000	1.094953000	-0.210418000
H	-2.623481000	3.992508000	2.295511000
H	-0.280480000	3.418567000	2.291931000
C	-1.057673000	-2.695192000	2.437758000
H	-1.905461000	-2.011197000	2.374320000
H	-1.296445000	-3.599572000	1.855454000
H	-0.939003000	-2.989916000	3.485830000
H	-0.041470000	-1.557699000	1.070461000
C	3.876181000	-0.036827000	2.369402000
H	4.779182000	-0.658719000	2.427811000
H	3.830075000	0.423250000	1.381388000
H	3.937207000	0.758360000	3.112400000
H	2.678927000	-1.197712000	3.532508000
H	-3.206958000	3.483095000	-0.097977000
H	-1.492189000	3.848311000	0.056685000

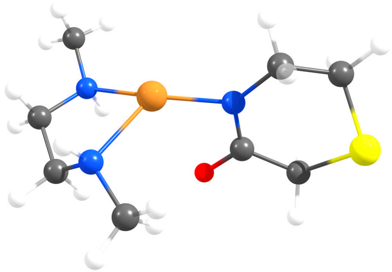

Figure S5: Lactam-copper complex substituted (X: S) computed structure in gas phase.

#p opt freq wB97XD/gen pseudo=read nosymm int=(grid=ultrafine)

32

Cu	-0.038207000	-0.558610000	2.297685000
N	2.247473000	-0.783141000	2.209291000
N	0.025541000	-2.629162000	2.231080000
C	-1.338009000	2.242376000	2.626411000
C	-0.569401000	1.235386000	0.517651000
C	-2.231272000	3.219567000	1.879125000
H	-1.927559000	1.783749000	3.428853000
C	-0.972695000	2.424105000	-0.351807000
S	-1.332577000	3.987287000	0.504211000
H	-3.120358000	2.716697000	1.486977000
H	-0.151520000	2.610078000	-1.042989000
N	-0.775764000	1.172952000	1.823346000
O	-0.055631000	0.276519000	-0.104777000
C	2.341372000	-2.103776000	1.597173000
H	2.017975000	-2.003959000	0.555780000

H	3.366916000	-2.501797000	1.584558000
C	1.422797000	-3.077433000	2.323721000
H	1.687718000	-3.113421000	3.386164000
H	1.547202000	-4.092374000	1.922540000
H	-1.838279000	2.118220000	-0.945804000
H	-2.553889000	4.024434000	2.542186000
H	-0.532420000	2.809755000	3.113101000
C	-0.881338000	-3.408782000	3.077981000
H	-1.906626000	-3.086011000	2.897555000
H	-0.804737000	-4.488081000	2.889442000
H	-0.645254000	-3.220759000	4.127012000
H	-0.272444000	-2.728498000	1.263527000
C	2.826432000	0.286721000	1.395924000
H	3.884505000	0.113366000	1.151769000
H	2.240885000	0.373022000	0.478614000
H	2.734399000	1.229971000	1.936231000
H	2.694983000	-0.801290000	3.119080000

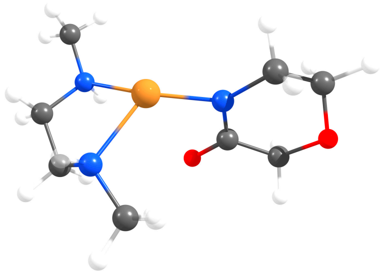

Figure S6: Lactam-copper complex substituted (X: O) computed structure in gas phase

#p opt freq wB97XD/gen pseudo=read nosymm int=(grid=ultrafine)

32

Cu	-0.015956000	-0.552973000	2.379458000
N	2.263384000	-0.801527000	2.208449000
N	0.020739000	-2.623058000	2.256056000
C	-1.359812000	2.275468000	2.612221000
C	-0.560166000	1.250161000	0.586844000
C	-2.181398000	3.160122000	1.692950000
H	-2.001375000	1.879874000	3.407423000
C	-0.994522000	2.491331000	-0.184676000
O	-1.389406000	3.590027000	0.608489000
H	-3.062515000	2.608894000	1.325455000
H	-0.154492000	2.817312000	-0.798807000
N	-0.749547000	1.170036000	1.894766000
O	-0.047142000	0.323357000	-0.078858000
C	2.312993000	-2.098816000	1.542959000

H	1.944311000	-1.954160000	0.522105000
H	3.331928000	-2.507198000	1.469803000
C	1.415257000	-3.088772000	2.273198000
H	1.724511000	-3.165876000	3.321438000
H	1.510450000	-4.089806000	1.831229000
H	-1.807626000	2.185603000	-0.861728000
H	-2.524544000	4.057948000	2.211189000
H	-0.589113000	2.890646000	3.097981000
C	-0.858619000	-3.419999000	3.115484000
H	-1.886753000	-3.080744000	2.989474000
H	-0.803109000	-4.493328000	2.889398000
H	-0.576071000	-3.268986000	4.159049000
H	-0.319049000	-2.687491000	1.299305000
C	2.832200000	0.287960000	1.413769000
H	3.879322000	0.107697000	1.130344000
H	2.219348000	0.412473000	0.518774000
H	2.772355000	1.213011000	1.988981000
H	2.744636000	-0.862070000	3.099090000

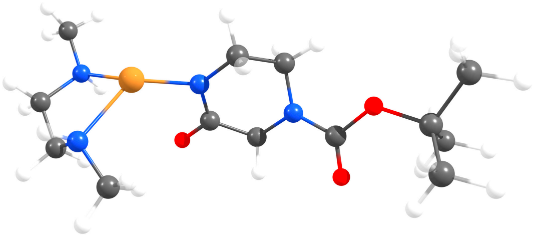

Figure S7: Lactam-copper complex substituted (X: *N*-Boc) computed structure in gas phase.

#p opt freq wB97XD/gen pseudo=read nosymm int=(grid=ultrafine)

48

Cu	-0.090798000	-0.527046000	2.326369000
N	2.155543000	-0.891359000	2.608613000
N	-0.125083000	-2.588459000	2.088600000
C	-1.320194000	2.357098000	2.428874000
C	-0.179416000	1.356010000	0.546694000
C	-1.989400000	3.288368000	1.430404000
H	-2.078018000	1.957521000	3.110345000
C	-0.345183000	2.633407000	-0.267852000
N	-1.004281000	3.708151000	0.452257000
H	-2.803617000	2.759004000	0.917003000
H	0.643376000	2.967242000	-0.580280000
N	-0.638777000	1.251340000	1.784887000
O	0.410324000	0.413070000	-0.025847000
C	2.285208000	-2.154658000	1.889532000
H	2.141607000	-1.938310000	0.825856000

H	3.278860000	-2.613251000	2.001214000
C	1.214528000	-3.130139000	2.359854000
H	1.297623000	-3.278446000	3.442247000
H	1.355804000	-4.110560000	1.885231000
H	-0.896003000	2.369321000	-1.179331000
H	-2.401932000	4.163935000	1.924112000
H	-0.619247000	2.944680000	3.039880000
C	-1.196472000	-3.380279000	2.698122000
H	-2.160768000	-2.978479000	2.387080000
H	-1.142012000	-4.441423000	2.420200000
H	-1.129493000	-3.301324000	3.784862000
H	-0.262006000	-2.581291000	1.080505000
C	2.927153000	0.201472000	2.014449000
H	4.001117000	-0.023593000	1.944338000
H	2.523644000	0.401439000	1.019992000
H	2.789877000	1.100219000	2.617425000
H	2.439148000	-1.023732000	3.573324000
C	-0.694994000	4.992662000	0.142609000
O	0.124436000	5.307633000	-0.695865000
O	-1.409712000	5.862429000	0.888063000
C	-1.237170000	7.297675000	0.731757000
C	0.190752000	7.704488000	1.088421000
H	0.448279000	7.333053000	2.083941000
H	0.900369000	7.305080000	0.365902000
H	0.266985000	8.795242000	1.099142000
C	-1.620492000	7.729698000	-0.681762000
H	-0.922294000	7.327156000	-1.413666000
H	-2.628221000	7.379217000	-0.920226000
H	-1.613607000	8.821390000	-0.743429000
C	-2.222312000	7.865153000	1.748214000
H	-3.239233000	7.540265000	1.516373000
H	-1.969878000	7.525068000	2.755288000
H	-2.192071000	8.957278000	1.730727000

Aryl Halides

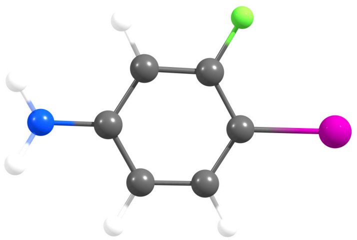

Figure S8: 3-fluoro-4-iodoaniline computed structure in gas phase.

#p opt freq wB97XD/gen pseudo=read nosymm int=(grid=ultrafine)

14

C	0.337491000	2.884378000	-2.899233000
C	1.298521000	2.001468000	-2.401792000
C	-1.002687000	2.477203000	-2.908703000
H	-1.766205000	3.142460000	-3.296200000
C	0.917369000	0.758803000	-1.932326000
C	-1.362449000	1.228115000	-2.433630000
H	-2.403799000	0.931610000	-2.448654000
C	-0.407141000	0.348751000	-1.937276000
N	0.711149000	4.110891000	-3.421506000
H	1.594099000	4.476644000	-3.105251000
H	-0.011953000	4.810068000	-3.461984000
I	-0.953651000	-1.554627000	-1.214875000
H	2.349845000	2.263282000	-2.378356000
F	1.871711000	-0.050251000	-1.464790000

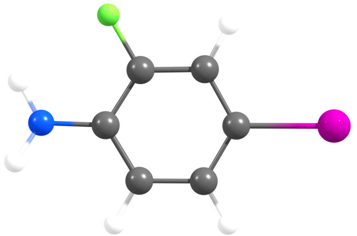

Figure S9: 2-fluoro-4-iodoaniline computed structure in gas phase.

#p opt freq wB97XD/gen pseudo=read nosymm int=(grid=ultrafine)

14

C	0.299261000	3.118238000	-2.608070000
C	1.236248000	2.156705000	-2.224469000
C	-1.040379000	2.727746000	-2.591097000
H	-1.801525000	3.441247000	-2.887945000
C	0.894559000	0.878411000	-1.842865000
C	-1.416668000	1.446126000	-2.210086000
H	-2.464547000	1.175831000	-2.208078000
C	-0.449409000	0.525116000	-1.838969000
N	0.715237000	4.367668000	-3.034237000
H	1.641823000	4.633299000	-2.741974000
H	0.039552000	5.107291000	-2.936604000
I	-1.013979000	-1.432327000	-1.254057000
F	2.536608000	2.521703000	-2.223708000
H	1.677134000	0.188807000	-1.555478000

Transition states (TS)

3-fluoro-4-iodoaniline

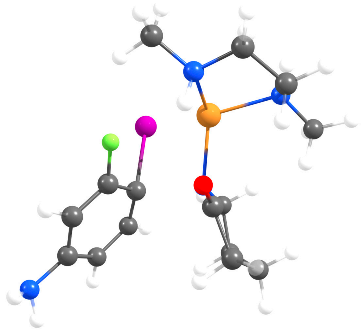

Figure S10: Lactam-copper complex unsubstituted (X: CH_2) transition state computed structure in gas phase.

#p opt=(calcfc,ts,tight,noeigentest) freq wB97XD/gen pseudo=read nosymm int=(grid=ultrafine)

48

Cu	-1.250862000	-1.409042000	0.026397000
N	0.905022000	-1.666030000	0.076755000
N	-1.099757000	-1.973670000	2.125444000
C	-3.367242000	0.746634000	0.227678000
C	-1.053474000	1.473431000	0.516917000
C	-3.857091000	2.157274000	0.536832000
H	-3.922819000	0.310525000	-0.608907000
C	-1.536388000	2.789993000	1.106097000
C	-2.974119000	2.775457000	1.611189000
H	-3.815851000	2.779658000	-0.362224000
H	-0.820162000	3.091834000	1.871526000
N	-1.938955000	0.600288000	-0.072108000
O	0.154598000	1.253853000	0.504090000
C	1.249052000	-2.142366000	1.410328000
H	1.185462000	-3.237608000	1.403643000
H	2.278452000	-1.884805000	1.699662000
C	0.281557000	-1.582983000	2.443515000
H	0.326521000	-0.490216000	2.421295000
H	0.574789000	-1.914211000	3.450160000
H	-1.447020000	3.511177000	0.282368000

H	-4.902252000	2.110045000	0.857106000
H	-3.602892000	0.103595000	1.086146000
C	-0.986003000	3.182686000	-3.589574000
C	-0.031159000	2.259077000	-3.165252000
C	-2.322791000	2.915114000	-3.297151000
H	-3.100768000	3.581715000	-3.654778000
C	-0.404296000	1.141768000	-2.443870000
C	-2.685404000	1.782908000	-2.575761000
H	-3.734393000	1.585794000	-2.395790000
C	-1.727288000	0.901302000	-2.103085000
N	-0.615560000	4.290978000	-4.363505000
H	0.325165000	4.611875000	-4.193032000
H	-1.268466000	5.057836000	-4.312785000
I	-2.356771000	-1.524207000	-2.383163000
H	1.019980000	2.381340000	-3.401945000
F	0.556878000	0.258067000	-2.126738000
C	-2.072258000	-1.340645000	3.014140000
H	-3.074749000	-1.694761000	2.769883000
H	-1.870981000	-1.539446000	4.076134000
H	-2.044858000	-0.260219000	2.854051000
H	-1.182297000	-2.981459000	2.212102000
H	-3.042837000	2.181754000	2.531604000
H	-3.299118000	3.788389000	1.863832000
C	1.595177000	-2.377673000	-0.991903000
H	2.689708000	-2.323866000	-0.900705000
H	1.297626000	-3.429659000	-0.976703000
H	1.295804000	-1.956137000	-1.949511000
H	1.070227000	-0.661641000	0.010151000

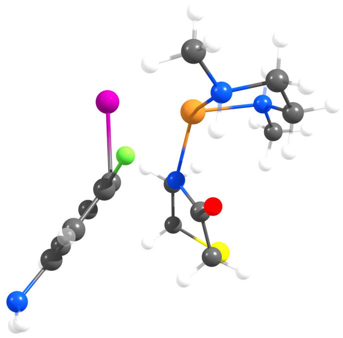

Figure S11: Lactam-copper complex substituted (X: S) transition state computed structure in gas phase.

#p opt=(calcfc,ts,tight,noeigentest) freq wB97XD/gen pseudo=read nosymm int=(grid=ultrafine)

46

Cu	-1.247985000	-1.421687000	0.007466000
N	0.903658000	-1.663036000	0.066477000
N	-1.109173000	-1.941104000	2.114224000
C	-3.366359000	0.709061000	0.211642000
C	-1.039875000	1.469330000	0.496674000
C	-3.939369000	2.077835000	0.542593000
H	-3.901083000	0.283508000	-0.645176000
C	-1.463169000	2.783400000	1.143397000
S	-3.076919000	2.797912000	1.967064000
H	-3.856076000	2.770248000	-0.298014000
H	-0.700604000	3.059541000	1.868970000
N	-1.936560000	0.616055000	-0.091166000
O	0.168664000	1.266580000	0.429123000
C	1.243056000	-2.120488000	1.408776000
H	1.179788000	-3.215618000	1.416603000
H	2.271452000	-1.858649000	1.697008000
C	0.271971000	-1.548358000	2.431798000
H	0.318225000	-0.455963000	2.401298000
H	0.561388000	-1.869310000	3.442664000
H	-1.461805000	3.534305000	0.344957000
H	-4.992926000	1.982232000	0.810883000
H	-3.586348000	0.037884000	1.048812000
C	-0.968596000	3.182456000	-3.600584000
C	-0.022087000	2.243844000	-3.190897000
C	-2.305753000	2.933312000	-3.291917000
H	-3.078618000	3.611526000	-3.638278000
C	-0.400552000	1.130656000	-2.465570000
C	-2.674962000	1.805324000	-2.567741000
H	-3.724758000	1.622417000	-2.377382000
C	-1.722913000	0.909379000	-2.109706000
N	-0.592871000	4.286263000	-4.376032000
H	0.355364000	4.592714000	-4.222287000
H	-1.234265000	5.062240000	-4.319874000
I	-2.368110000	-1.520909000	-2.392320000
H	1.027021000	2.350073000	-3.443582000
F	0.550980000	0.230976000	-2.167409000
C	-2.082297000	-1.305387000	3.003614000
H	-3.082959000	-1.670418000	2.767750000
H	-1.873914000	-1.499228000	4.064808000
H	-2.068400000	-0.224987000	2.841570000
H	-1.188804000	-2.948426000	2.210469000
C	1.595316000	-2.392645000	-0.989809000
H	2.689469000	-2.339904000	-0.895916000
H	1.295222000	-3.443464000	-0.959608000
H	1.299780000	-1.984668000	-1.954535000
H	1.075846000	-0.661242000	-0.015717000

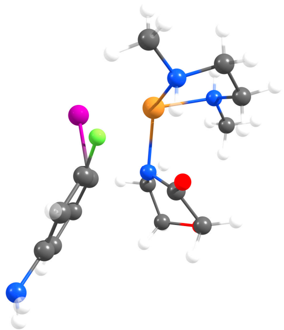

Figure S12: Lactam-copper complex substituted (X: O) transition state computed structure in gas phase.

#p opt=(calcfc,ts,tight,noeigentest) freq wB97XD/gen pseudo=read nosymm int=(grid=ultrafine)

46

Cu	-1.271360000	-1.472082000	-0.059926000
N	0.879119000	-1.585410000	0.050303000
N	-1.148674000	-1.859143000	2.085864000
C	-3.351882000	0.716666000	0.164523000
C	-1.052790000	1.399242000	0.452856000
C	-3.710557000	2.074190000	0.728142000
H	-3.954860000	0.490405000	-0.720179000
C	-1.546309000	2.457866000	1.438983000
O	-2.887370000	2.342368000	1.846680000
H	-3.580876000	2.857142000	-0.034882000
H	-0.922247000	2.412822000	2.332137000
N	-1.936778000	0.590004000	-0.187903000
O	0.160517000	1.357395000	0.254784000
C	1.221804000	-1.952970000	1.419966000
H	1.218179000	-3.047978000	1.485304000
H	2.229720000	-1.619391000	1.705453000
C	0.207149000	-1.383522000	2.400869000
H	0.202904000	-0.292756000	2.321373000
H	0.496393000	-1.642146000	3.429407000
H	-1.367746000	3.429952000	0.952164000
H	-4.742832000	2.093409000	1.081334000
H	-3.616262000	-0.050985000	0.899702000
C	-0.960819000	3.134304000	-3.677642000
C	-0.028005000	2.174576000	-3.287119000
C	-2.299248000	2.905127000	-3.361888000
H	-3.062025000	3.603317000	-3.690531000
C	-0.421005000	1.060232000	-2.573260000
C	-2.685292000	1.772682000	-2.653064000
H	-3.737524000	1.611985000	-2.456859000

C	-1.744799000	0.855616000	-2.212778000
N	-0.570284000	4.243172000	-4.440242000
H	0.382243000	4.533158000	-4.280057000
H	-1.198731000	5.028341000	-4.365776000
I	-2.427050000	-1.543495000	-2.439078000
H	1.022333000	2.269421000	-3.538717000
F	0.516438000	0.144015000	-2.283083000
C	-2.161795000	-1.236655000	2.940628000
H	-3.137860000	-1.672387000	2.722397000
H	-1.945952000	-1.366308000	4.009952000
H	-2.214445000	-0.166771000	2.726371000
H	-1.177228000	-2.863692000	2.229314000
C	1.626767000	-2.336984000	-0.952598000
H	2.714248000	-2.215057000	-0.848578000
H	1.387211000	-3.399708000	-0.861098000
H	1.324247000	-2.005014000	-1.943660000
H	1.012422000	-0.581376000	-0.084467000

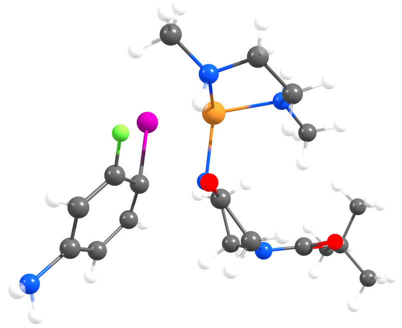

Figure S13: Lactam-copper complex substituted (X: *N*-Boc) transition state computed structure in gas phase.

#p opt=(calcfc,ts,tight,noeigentest) freq wB97XD/gen pseudo=read nosymm int=(grid=ultrafine)

62

Cu	-1.318847000	0.388688000	-1.303128000
N	0.819600000	0.156521000	-1.068344000
N	-1.361796000	-0.282296000	0.761427000
C	-3.345132000	2.632134000	-1.046733000
C	-1.042958000	3.190236000	-0.504887000
C	-3.728212000	3.994886000	-0.495607000
H	-3.865996000	2.421855000	-1.985431000
C	-1.546622000	4.215678000	0.514883000
N	-2.978455000	4.201087000	0.735225000
H	-3.450105000	4.786740000	-1.198431000
H	-1.035669000	4.046584000	1.460177000
N	-1.908585000	2.471901000	-1.275543000

O	0.176398000	3.089611000	-0.613874000
C	1.047921000	-0.385764000	0.266838000
H	1.008860000	-1.479744000	0.195746000
H	2.040376000	-0.127283000	0.662667000
C	-0.021539000	0.096169000	1.236145000
H	0.010459000	1.187684000	1.299249000
H	0.178174000	-0.300860000	2.241310000
H	-1.234122000	5.195910000	0.136559000
H	-4.794399000	4.059638000	-0.296419000
H	-3.694019000	1.866896000	-0.342943000
H	-1.428708000	-1.295308000	0.758458000
C	1.605522000	-0.509124000	-2.101968000
H	2.686893000	-0.455978000	-1.911565000
H	1.314839000	-1.561697000	-2.155149000
H	1.390257000	-0.049316000	-3.064252000
C	-0.592455000	5.302366000	-4.427662000
C	0.282231000	4.281127000	-4.059082000
C	-1.957177000	5.088003000	-4.238893000
H	-2.674145000	5.836185000	-4.560619000
C	-0.193103000	3.119282000	-3.484027000
C	-2.425490000	3.908762000	-3.669440000
H	-3.493555000	3.762943000	-3.571302000
C	-1.545764000	2.926769000	-3.244114000
N	-0.115487000	6.462835000	-5.051042000
H	0.829622000	6.704719000	-4.795624000
H	-0.726789000	7.258570000	-4.951087000
I	-2.269760000	0.576348000	-3.765810000
H	1.350886000	4.363573000	-4.222587000
F	0.694619000	2.148799000	-3.217977000
H	1.000826000	1.161679000	-1.073869000
C	-2.418551000	0.254793000	1.619511000
H	-3.392989000	-0.039277000	1.226784000
H	-2.334755000	-0.085118000	2.660549000
H	-2.358750000	1.344467000	1.622865000
C	-3.442079000	3.802978000	1.956522000
O	-2.739217000	3.654994000	2.935806000
O	-4.771437000	3.609047000	1.925901000
C	-5.500151000	3.190794000	3.117306000
C	-5.374344000	4.243932000	4.214367000
H	-6.035929000	3.982974000	5.044617000
H	-4.351426000	4.306690000	4.581565000
H	-5.676029000	5.221998000	3.831041000
C	-6.937070000	3.106581000	2.615715000
H	-7.016572000	2.383536000	1.800632000
H	-7.598391000	2.792116000	3.426416000
H	-7.271606000	4.080040000	2.250621000
C	-5.012799000	1.818679000	3.576017000
H	-5.077449000	1.103775000	2.751150000

H	-3.983540000	1.867970000	3.928515000
H	-5.649470000	1.459151000	4.388476000

2-fluoro-4-iodoaniline

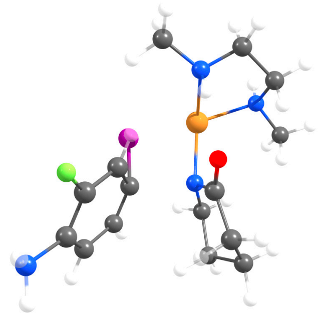

Figure S14: Lactam-copper complex usubstituted (X:CH_2) transition state computed structure in gas phase.

#p opt=(calcfc,ts,tight,noeigentest) freq wB97XD/gen pseudo=read nosymm int=(grid=ultrafine)

48

Cu	-1.289209000	-1.384360000	0.103027000
N	0.896053000	-1.570158000	0.119894000
N	-1.120122000	-2.117592000	2.113008000
C	-3.378148000	0.815803000	0.313496000
C	-1.050579000	1.497450000	0.557058000
C	-3.834014000	2.256386000	0.523162000
H	-3.944976000	0.337225000	-0.492161000
C	-1.489468000	2.876952000	1.014579000
C	-2.922092000	2.940413000	1.532166000
H	-3.796008000	2.805256000	-0.422498000
H	-0.758749000	3.236643000	1.739967000
N	-1.955520000	0.618934000	0.020053000
O	0.152182000	1.231914000	0.580044000
C	1.228816000	-2.228410000	1.376019000
H	1.145314000	-3.311873000	1.225423000
H	2.261424000	-2.028784000	1.699339000
C	0.267083000	-1.785846000	2.470766000
H	0.324866000	-0.698289000	2.578295000
H	0.556815000	-2.234374000	3.431472000
H	-1.394860000	3.509544000	0.121012000
H	-4.874354000	2.256309000	0.862033000
H	-3.620965000	0.238274000	1.216336000
C	-1.011608000	3.106893000	-3.628597000
C	-0.069676000	2.241861000	-3.075584000

C	-2.346693000	2.811370000	-3.364241000
H	-3.121655000	3.433945000	-3.799943000
C	-0.394541000	1.151983000	-2.295886000
C	-2.720054000	1.717642000	-2.586981000
H	-3.768191000	1.497771000	-2.436856000
C	-1.733801000	0.910642000	-2.050918000
N	-0.614498000	4.155277000	-4.466791000
H	0.350547000	4.420505000	-4.340808000
H	-1.222054000	4.958795000	-4.425285000
I	-2.363571000	-1.551407000	-2.295550000
C	-2.090933000	-1.535867000	3.040059000
H	-3.095883000	-1.853654000	2.759352000
H	-1.903542000	-1.818892000	4.085138000
H	-2.044611000	-0.447249000	2.960736000
H	-1.226060000	-3.126787000	2.115584000
H	-2.991867000	2.428966000	2.500498000
H	-3.220236000	3.978763000	1.700389000
C	1.622410000	-2.088961000	-1.030215000
H	2.713778000	-2.020262000	-0.915929000
H	1.353419000	-3.136625000	-1.188142000
H	1.324753000	-1.540794000	-1.925096000
H	1.021827000	-0.561508000	0.215755000
F	1.235708000	2.501267000	-3.316514000
H	0.394455000	0.550604000	-1.870930000

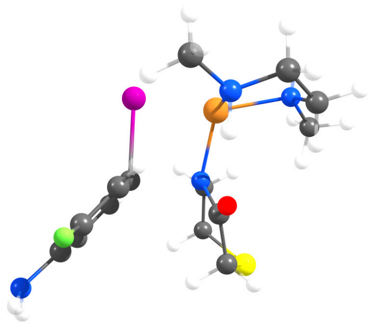

Figure S15: Lactam-copper complex substituted (X:S) transition state computed structure in gas phase

#p opt=(calcfc,ts,tight,noeigentest) freq wB97XD/gen pseudo=read nosymm int=(grid=ultrafine)

46

Cu	-1.279623000	-1.387612000	0.083716000
N	0.898549000	-1.586949000	0.108087000
N	-1.125403000	-2.058840000	2.116653000
C	-3.376388000	0.786840000	0.292275000

C	-1.034342000	1.497940000	0.536974000
C	-3.917137000	2.191190000	0.513847000
H	-3.919735000	0.309089000	-0.531409000
C	-1.414555000	2.891631000	1.011093000
S	-3.015215000	3.021591000	1.851692000
H	-3.838920000	2.804328000	-0.386232000
H	-0.637026000	3.245128000	1.685039000
N	-1.950212000	0.637115000	-0.001146000
O	0.166422000	1.228138000	0.535676000
C	1.219796000	-2.230556000	1.375841000
H	1.113401000	-3.314264000	1.243696000
H	2.257050000	-2.046562000	1.692850000
C	0.269668000	-1.748306000	2.463954000
H	0.349114000	-0.660495000	2.552495000
H	0.553026000	-2.184277000	3.432075000
H	-1.421019000	3.534465000	0.122786000
H	-4.966671000	2.140207000	0.808840000
H	-3.606452000	0.188870000	1.181526000
C	-0.994504000	3.119015000	-3.639793000
C	-0.057040000	2.243234000	-3.095368000
C	-2.330646000	2.835922000	-3.366592000
H	-3.102712000	3.466071000	-3.796238000
C	-0.386751000	1.156055000	-2.313665000
C	-2.709118000	1.745309000	-2.587149000
H	-3.758591000	1.533843000	-2.434306000
C	-1.726395000	0.928575000	-2.059458000
N	-0.593213000	4.163344000	-4.478808000
H	0.374550000	4.421752000	-4.360850000
H	-1.196045000	4.970445000	-4.441392000
I	-2.367575000	-1.540582000	-2.307316000
C	-2.080297000	-1.433462000	3.034172000
H	-3.092021000	-1.744093000	2.770039000
H	-1.889767000	-1.693143000	4.084395000
H	-2.019743000	-0.347745000	2.928270000
H	-1.251815000	-3.065259000	2.148309000
C	1.617422000	-2.135018000	-1.033793000
H	2.709432000	-2.084274000	-0.918033000
H	1.329764000	-3.179636000	-1.177361000
H	1.330850000	-1.593441000	-1.936373000
H	1.045675000	-0.580727000	0.188753000
F	1.248228000	2.487996000	-3.347755000
H	0.398846000	0.542699000	-1.899720000

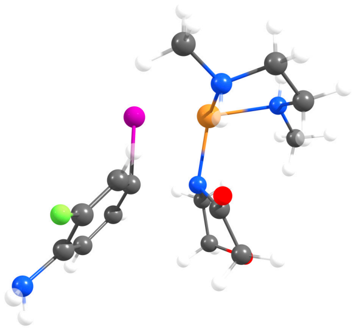

Figure S16: Lactam-copper complex substituted (X:O) transition state computed structure in gas phase

#p opt=(calcfc,ts,tight,noeigentest) freq wB97XD/gen pseudo=read nosymm int=(grid=ultrafine)

46

Cu	-1.311308000	-1.454240000	0.017871000
N	0.867546000	-1.523235000	0.058233000
N	-1.134300000	-1.977213000	2.096368000
C	-3.378428000	0.787964000	0.242730000
C	-1.072803000	1.421819000	0.531944000
C	-3.719195000	2.206830000	0.645696000
H	-3.975326000	0.468091000	-0.617151000
C	-1.554612000	2.622427000	1.341447000
O	-2.903289000	2.587779000	1.736426000
H	-3.569465000	2.896642000	-0.198416000
H	-0.945073000	2.699677000	2.241574000
N	-1.963507000	0.597989000	-0.079402000
O	0.147627000	1.286947000	0.416311000
C	1.218838000	-2.077188000	1.360785000
H	1.168370000	-3.170758000	1.291196000
H	2.244714000	-1.821770000	1.664477000
C	0.245456000	-1.582273000	2.421903000
H	0.275298000	-0.488936000	2.450189000
H	0.548115000	-1.950436000	3.411941000
H	-1.350418000	3.507939000	0.716765000
H	-4.754311000	2.281520000	0.982927000
H	-3.655884000	0.115223000	1.062297000
C	-0.976926000	3.060197000	-3.690163000
C	-0.057186000	2.150098000	-3.173751000
C	-2.315981000	2.807447000	-3.404808000
H	-3.076565000	3.465377000	-3.812732000
C	-0.405131000	1.060361000	-2.404876000
C	-2.714428000	1.713501000	-2.640024000
H	-3.768052000	1.532348000	-2.478381000
C	-1.747197000	0.864395000	-2.134309000

N	-0.557896000	4.110360000	-4.514356000
H	0.418275000	4.338391000	-4.401999000
H	-1.135321000	4.934000000	-4.446096000
I	-2.441124000	-1.572909000	-2.348497000
C	-2.115029000	-1.340493000	2.978853000
H	-3.111049000	-1.719030000	2.745724000
H	-1.904509000	-1.519631000	4.041821000
H	-2.112869000	-0.262468000	2.801416000
H	-1.214241000	-2.984260000	2.193518000
C	1.622240000	-2.098959000	-1.047286000
H	2.708293000	-1.971509000	-0.936407000
H	1.402557000	-3.167129000	-1.120783000
H	1.305366000	-1.637549000	-1.983610000
H	0.970202000	-0.506090000	0.084751000
F	1.251410000	2.363752000	-3.438533000
H	0.369984000	0.420554000	-2.015285000

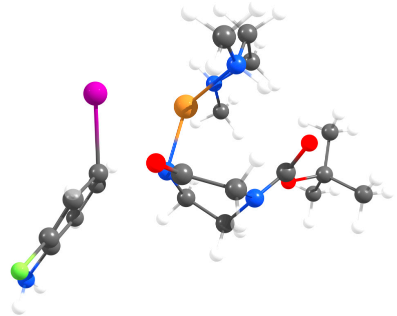

Figure S17: Lactam-copper complex substituted (X:*N*-Boc) transition state computed structure in gas phase

#p opt=(calcfc,ts,tight,noeigentest) freq wB97XD/gen pseudo=read nosymm int=(grid=ultrafine)

62

Cu	-0.939326000	0.112557000	0.317011000
N	0.108375000	0.388179000	2.168686000
N	-2.268466000	-1.101266000	1.491128000
C	-2.686545000	2.050414000	-1.068495000
C	-0.321672000	2.461022000	-0.595445000
C	-2.936814000	3.351479000	-0.313458000
H	-3.020394000	2.176875000	-2.106494000
C	-0.702611000	3.439939000	0.526260000
N	-2.103527000	3.374022000	0.878099000
H	-2.645854000	4.212289000	-0.923688000
H	-0.086968000	3.212792000	1.394560000
N	-1.283717000	1.623785000	-1.083916000
O	0.857123000	2.451805000	-0.940466000
C	-0.338447000	-0.744731000	2.983485000

H	0.144848000	-1.651024000	2.602570000
H	-0.050350000	-0.632882000	4.037789000
C	-1.850839000	-0.872314000	2.882112000
H	-2.301326000	0.067692000	3.208334000
H	-2.212209000	-1.664264000	3.550312000
H	-0.451292000	4.451731000	0.190882000
H	-3.985899000	3.454170000	-0.050215000
H	-3.301678000	1.256241000	-0.635696000
C	-1.730778000	1.801987000	-5.476866000
C	-0.525147000	2.171861000	-4.882530000
C	-2.498531000	0.884652000	-4.763490000
H	-3.437257000	0.541825000	-5.188438000
C	-0.072096000	1.688181000	-3.673648000
C	-2.090330000	0.376446000	-3.531958000
H	-2.712727000	-0.344767000	-3.017614000
C	-0.884730000	0.797226000	-2.980455000
N	-2.086521000	2.273661000	-6.750257000
H	-1.688804000	3.179136000	-6.960055000
H	-3.087294000	2.297022000	-6.890662000
I	0.267563000	-1.010872000	-1.884267000
C	-2.496359000	2.939799000	2.100733000
O	-1.735951000	2.766779000	3.042053000
O	-3.822436000	2.737371000	2.143701000
C	-4.573367000	2.819969000	3.400187000
C	-4.275494000	4.148253000	4.085980000
H	-4.942844000	4.269617000	4.942907000
H	-3.245654000	4.189495000	4.442530000
H	-4.447716000	4.980060000	3.397273000
C	-6.019305000	2.769883000	2.926001000
H	-6.213709000	1.840631000	2.384069000
H	-6.693264000	2.816322000	3.784806000
H	-6.238824000	3.612185000	2.265345000
C	-4.267580000	1.631702000	4.302861000
H	-4.462079000	0.692315000	3.779477000
H	-3.231355000	1.648777000	4.638312000
H	-4.920848000	1.672762000	5.178857000
C	-3.702922000	-0.886319000	1.292689000
H	-3.966726000	-1.138481000	0.263969000
H	-4.317767000	-1.492214000	1.972513000
H	-3.934594000	0.168114000	1.461310000
H	-2.050728000	-2.062414000	1.245032000
H	-0.277727000	1.239187000	2.576661000
C	1.560491000	0.516659000	2.077743000
H	2.037550000	0.626210000	3.061419000
H	1.973113000	-0.367975000	1.586097000
H	1.802780000	1.384185000	1.461165000
F	0.248951000	3.070789000	-5.545978000
H	0.860689000	2.040291000	-3.256762000

Isolated Products

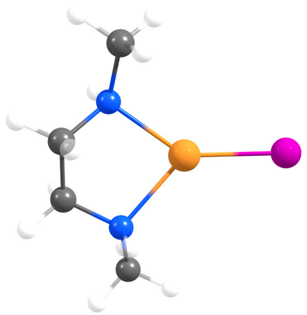

Figure S18: Activated copper catalyst computed structure in gas phase.

#p opt freq wB97XD/gen pseudo=read nosymm int=(grid=ultrafine)

20

Cu	-0.388291000	-0.293763000	0.985767000
N	1.340475000	-1.569942000	0.957778000
N	-1.343146000	-2.097202000	1.796683000
C	0.854237000	-2.943012000	1.127641000
H	0.452254000	-3.271932000	0.163308000
H	1.660229000	-3.639429000	1.395672000
C	-0.246153000	-2.985610000	2.181354000
H	0.149047000	-2.627947000	3.138479000
H	-0.575053000	-4.022268000	2.337361000
I	-1.215563000	1.985500000	0.499429000
C	-2.304416000	-1.833299000	2.869413000
H	-3.089996000	-1.181750000	2.486670000
H	-2.753093000	-2.750571000	3.273804000
H	-1.799849000	-1.302025000	3.678803000
H	-1.836870000	-2.501211000	1.007249000
C	2.211196000	-1.396178000	-0.209161000
H	3.074414000	-2.074667000	-0.195533000
H	1.633840000	-1.579754000	-1.117054000
H	2.561233000	-0.364550000	-0.240112000
H	1.859106000	-1.303249000	1.789100000

3-fluoro-4-iodoaniline

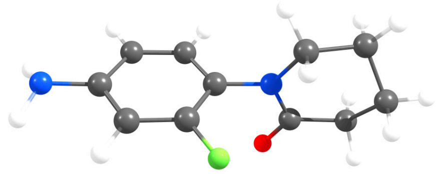

Figure S19: Coupled product substituted (X:CH_2) computed structure in gas phase.

#p opt freq wB97XD/6-311G(d,p) nosymm int=(grid=ultrafine)

28

C	-1.766598000	1.515621000	0.640170000
C	-0.121971000	3.365849000	1.125466000
C	-2.411701000	1.796588000	1.986350000
H	-2.525835000	1.447949000	-0.143283000
C	-0.580177000	3.366335000	2.574845000
C	-1.336264000	2.117813000	3.015434000
H	-3.101074000	2.643804000	1.897007000
H	0.311604000	3.547268000	3.176029000
N	-0.818715000	2.563336000	0.246729000
O	0.780342000	4.085982000	0.756355000
H	-1.219002000	4.249986000	2.688983000
H	-3.001140000	0.924765000	2.281694000
H	-1.243754000	0.550659000	0.673530000
C	0.582399000	2.325179000	-3.747910000
C	1.107489000	1.490901000	-2.756266000
C	-0.416454000	3.241686000	-3.397926000
H	-0.834128000	3.895529000	-4.155556000
C	0.637744000	1.599379000	-1.464283000
C	-0.867361000	3.321133000	-2.091353000
H	-1.629429000	4.041978000	-1.818973000
C	-0.352482000	2.500748000	-1.096590000
N	1.006872000	2.203809000	-5.064080000
H	1.926007000	1.810953000	-5.188121000
H	0.857629000	3.016898000	-5.639550000
H	1.879783000	0.763796000	-2.977791000
F	1.156789000	0.794748000	-0.522819000
H	-1.770621000	2.269840000	4.006705000
H	-0.644619000	1.271341000	3.094637000

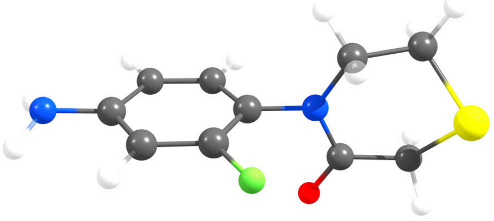

Figure S20: Coupled product substituted (X:S) computed structure in gas phase.

#p opt freq wB97XD/6-311G(d,p) nosymm int=(grid=ultrafine)

26

C	-1.734463000	1.516935000	0.632236000
C	-0.063986000	3.369101000	1.109563000
C	-2.458551000	1.785773000	1.934786000
H	-2.466697000	1.442718000	-0.177509000
C	-0.488027000	3.458225000	2.569173000
S	-1.282655000	2.000444000	3.295098000
H	-3.091420000	2.674599000	1.859287000
H	0.415250000	3.668687000	3.138742000
N	-0.786997000	2.571404000	0.252280000
O	0.843607000	4.070758000	0.721214000
H	-1.151076000	4.323793000	2.663755000
H	-3.092499000	0.932868000	2.180294000
H	-1.205198000	0.559662000	0.696555000
C	0.566746000	2.328683000	-3.759712000
C	1.105514000	1.496295000	-2.773576000
C	-0.429041000	3.244815000	-3.398314000
H	-0.857427000	3.896873000	-4.151350000
C	0.652641000	1.605013000	-1.475700000
C	-0.862885000	3.325631000	-2.086436000
H	-1.622393000	4.046120000	-1.805770000
C	-0.333613000	2.506659000	-1.098278000
N	0.974094000	2.206444000	-5.079689000
H	1.888270000	1.807446000	-5.218729000
H	0.814639000	3.015490000	-5.657851000
H	1.875675000	0.769724000	-3.003815000
F	1.182558000	0.803016000	-0.540504000

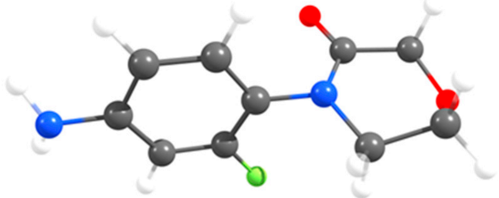

Figure S21: Coupled product substituted (X:O) computed structure in gas phase.

#p opt freq wB97XD/6-311G(d,p) nosymm int=(grid=ultrafine)

26

C	-1.592290000	1.448036000	0.740627000
C	-0.198362000	3.480324000	1.061778000

C	-2.233789000	1.851404000	2.051763000
H	-2.361342000	1.234400000	-0.007158000
C	-0.615951000	3.438049000	2.528176000
O	-1.239648000	2.254508000	2.962619000
H	-2.959918000	2.663180000	1.889294000
H	0.287700000	3.573135000	3.120921000
N	-0.753415000	2.536279000	0.237421000
O	0.549209000	4.351570000	0.675321000
H	-1.266177000	4.310852000	2.694997000
H	-2.754632000	1.002262000	2.495506000
H	-0.989600000	0.545437000	0.884464000
C	0.518948000	2.308218000	-3.799351000
C	1.026545000	1.427223000	-2.839924000
C	-0.418870000	3.269831000	-3.401439000
H	-0.820877000	3.962035000	-4.132867000
C	0.603022000	1.535409000	-1.531564000
C	-0.831856000	3.342878000	-2.083296000
H	-1.547319000	4.096348000	-1.776537000
C	-0.332884000	2.474778000	-1.119707000
N	0.897637000	2.192972000	-5.128289000
H	1.786759000	1.749260000	-5.291972000
H	0.771071000	3.021182000	-5.687114000
H	1.753180000	0.665714000	-3.097109000
F	1.114925000	0.691891000	-0.620811000

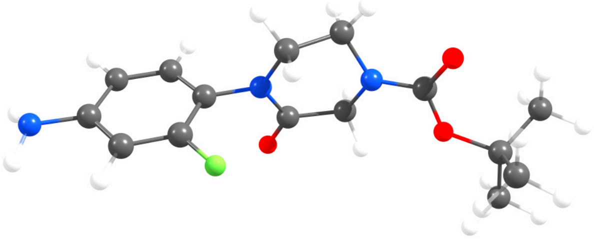

Figure S22: Coupled product substituted (X:*N*-Boc) computed structure in gas phase.

#p opt freq wB97XD/6-311G(d,p) nosymm int=(grid=ultrafine)

42

C	-2.214229000	2.340696000	0.420565000
C	-1.148104000	4.547191000	-0.074779000
C	-3.301012000	3.125326000	1.126260000
H	-2.659580000	1.603144000	-0.251695000
C	-1.948834000	5.161119000	1.067830000
N	-2.692798000	4.208839000	1.863588000
H	-4.013017000	3.528022000	0.393606000
H	-1.237802000	5.694344000	1.693236000
N	-1.374967000	3.230301000	-0.384222000

O	-0.375773000	5.246785000	-0.692053000
H	-2.611923000	5.910474000	0.615035000
H	-3.840036000	2.491046000	1.826973000
H	-1.600922000	1.808633000	1.156249000
C	1.110683000	1.381405000	-3.276246000
C	1.450723000	1.355251000	-1.920046000
C	-0.068121000	2.031482000	-3.663598000
H	-0.345056000	2.060875000	-4.711529000
C	0.626192000	1.975640000	-1.005246000
C	-0.876082000	2.638858000	-2.718588000
H	-1.779845000	3.153995000	-3.022090000
C	-0.548016000	2.620869000	-1.369285000
N	1.899878000	0.727029000	-4.209610000
H	2.861864000	0.586584000	-3.946982000
H	1.801206000	1.045847000	-5.159748000
H	2.350676000	0.863331000	-1.570590000
F	0.970245000	1.950719000	0.291758000
C	-2.911805000	4.328566000	3.206228000
O	-3.561596000	3.536701000	3.854302000
O	-2.312001000	5.427630000	3.686307000
C	-2.350632000	5.748828000	5.110342000
C	-3.788945000	6.003538000	5.551713000
H	-4.247934000	6.765116000	4.915967000
H	-4.381237000	5.091482000	5.499278000
H	-3.791933000	6.370539000	6.581441000
C	-1.530408000	7.031264000	5.182914000
H	-1.485221000	7.387126000	6.214812000
H	-0.511873000	6.855013000	4.829960000
H	-1.982641000	7.809869000	4.564676000
C	-1.686656000	4.638295000	5.919267000
H	-2.267907000	3.718757000	5.871681000
H	-0.680268000	4.445672000	5.538519000
H	-1.602949000	4.951960000	6.963071000

2-fluoro-4-iodoaniline

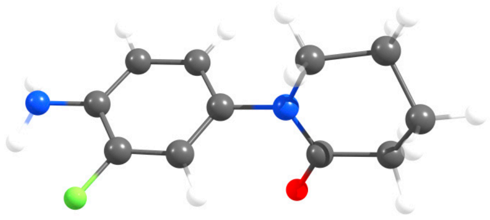

Figure S23: Coupled product unsubstituted (X:CH_2) computed structure in gas phase.

#p opt freq wB97XD/6-311G(d,p) nosymm int=(grid=ultrafine)

28
symmetry c1

C	-1.912728000	1.619138000	0.928839000
C	0.000313000	3.059909000	1.713556000
C	-2.568646000	1.829064000	2.282608000
H	-2.631123000	1.796545000	0.124837000
C	-0.522723000	2.942124000	3.136981000
C	-1.510637000	1.806498000	3.377029000
H	-3.089854000	2.792964000	2.294678000
H	0.355654000	2.873493000	3.779945000
N	-0.768843000	2.510729000	0.712022000
O	1.028811000	3.659461000	1.480769000
H	-0.997999000	3.904240000	3.361002000
H	-3.319252000	1.049038000	2.433597000
H	-1.578611000	0.576179000	0.834337000
C	0.594294000	2.678513000	-3.303462000
C	1.262786000	1.938946000	-2.325286000
C	-0.542031000	3.376983000	-2.894955000
H	-1.089093000	3.964938000	-3.624066000
C	0.846852000	1.872796000	-1.016942000
C	-0.971651000	3.334428000	-1.575403000
H	-1.845666000	3.898894000	-1.271321000
C	-0.287732000	2.581642000	-0.631917000
N	1.033475000	2.654482000	-4.619722000
H	1.995288000	2.376080000	-4.734089000
H	0.798291000	3.470572000	-5.160950000
H	-0.987216000	0.843475000	3.357247000
H	-1.963329000	1.902471000	4.367018000
F	2.375053000	1.270379000	-2.706826000
H	1.420090000	1.297736000	-0.301612000

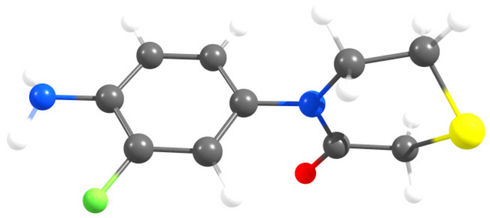

Figure S24: Coupled product substituted (X:S) computed structure in gas phase.

#p opt freq wB97XD/6-311G(d,p) nosymm int=(grid=ultrafine)

28

C	-1.912728000	1.619138000	0.928839000

C	0.000313000	3.059909000	1.713556000
C	-2.568646000	1.829064000	2.282608000
H	-2.631123000	1.796545000	0.124837000
C	-0.522723000	2.942124000	3.136981000
C	-1.510637000	1.806498000	3.377029000
H	-3.089854000	2.792964000	2.294678000
H	0.355654000	2.873493000	3.779945000
N	-0.768843000	2.510729000	0.712022000
O	1.028811000	3.659461000	1.480769000
H	-0.997999000	3.904240000	3.361002000
H	-3.319252000	1.049038000	2.433597000
H	-1.578611000	0.576179000	0.834337000
C	0.594294000	2.678513000	-3.303462000
C	1.262786000	1.938946000	-2.325286000
C	-0.542031000	3.376983000	-2.894955000
H	-1.089093000	3.964938000	-3.624066000
C	0.846852000	1.872796000	-1.016942000
C	-0.971651000	3.334428000	-1.575403000
H	-1.845666000	3.898894000	-1.271321000
C	-0.287732000	2.581642000	-0.631917000
N	1.033475000	2.654482000	-4.619722000
H	1.995288000	2.376080000	-4.734089000
H	0.798291000	3.470572000	-5.160950000
H	-0.987216000	0.843475000	3.357247000
H	-1.963329000	1.902471000	4.367018000
F	2.375053000	1.270379000	-2.706826000
H	1.420090000	1.297736000	-0.301612000

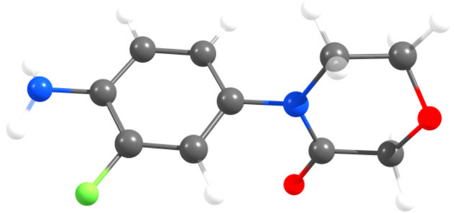

Figure 25
#p opt freq wB97XD/6-311G(d,p) nosymm int=(grid=ultrafine)

26

C	-1.833670000	1.394119000	0.946698000
C	0.042765000	2.803156000	1.752586000
C	-2.407900000	1.603971000	2.331880000
H	-2.597841000	1.593005000	0.190677000
C	-0.449996000	2.510011000	3.166586000
O	-1.389562000	1.471035000	3.293437000
H	-2.878944000	2.596692000	2.403626000

H	0.422605000	2.241315000	3.760345000
N	-0.703426000	2.295765000	0.721739000
O	1.027515000	3.492187000	1.595757000
H	-0.851333000	3.456391000	3.560913000
H	-3.160209000	0.844935000	2.548814000
H	-1.507813000	0.353530000	0.832318000
C	0.564235000	2.774539000	-3.298792000
C	1.346771000	2.113725000	-2.348718000
C	-0.656817000	3.286434000	-2.859080000
H	-1.293570000	3.809373000	-3.564373000
C	0.961787000	1.947073000	-1.040054000
C	-1.057861000	3.144228000	-1.537819000
H	-1.999672000	3.570678000	-1.211730000
C	-0.259795000	2.470040000	-0.624514000
N	0.984853000	2.853122000	-4.617540000
H	1.974444000	2.719038000	-4.752196000
H	0.621782000	3.635792000	-5.136732000
F	2.539299000	1.627730000	-2.759588000
H	1.621915000	1.440292000	-0.348814000

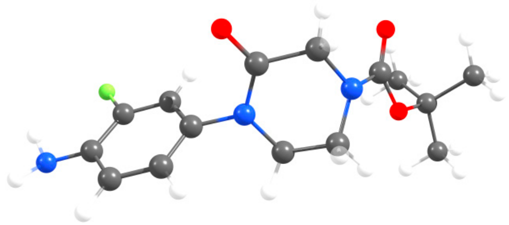

Figure S26: Coupled product substituted (X:*N*-Boc) computed structure in gas phase.

#p opt freq wB97XD/6-311G(d,p) nosymm int=(grid=ultrafine)

42

C	-1.321591000	4.223235000	1.195473000
C	0.673406000	2.835493000	1.811065000
C	-1.080588000	4.960452000	2.499148000
H	-1.442452000	4.936630000	0.376032000
C	0.501736000	3.286947000	3.258021000
N	-0.725788000	4.003286000	3.525620000
H	-0.250850000	5.667510000	2.384372000
H	0.540145000	2.398488000	3.884969000
N	-0.195503000	3.343993000	0.876695000
O	1.575587000	2.078136000	1.529051000
H	1.374550000	3.903904000	3.502796000
H	-1.967936000	5.512236000	2.795413000
H	-2.246618000	3.637439000	1.267652000
C	-0.028486000	1.942954000	-3.124029000

C	-0.425751000	1.121146000	-2.066384000
C	0.326857000	3.253350000	-2.802360000
H	0.643668000	3.922625000	-3.594881000
C	-0.483710000	1.550031000	-0.761910000
C	0.288824000	3.702860000	-1.489612000
H	0.589313000	4.717850000	-1.256005000
C	-0.120721000	2.860745000	-0.466192000
N	-0.049902000	1.464927000	-4.425209000
H	-0.033949000	0.460514000	-4.504676000
H	0.589466000	1.917609000	-5.057873000
F	-0.757755000	-0.154654000	-2.364943000
H	-0.783615000	0.860235000	0.016257000
C	-1.595903000	3.513992000	4.461552000
O	-1.331086000	2.603467000	5.213627000
O	-2.757065000	4.188672000	4.429523000
C	-3.832381000	3.880444000	5.368839000
C	-3.367314000	4.123983000	6.801524000
H	-4.218269000	4.021797000	7.479992000
H	-2.597642000	3.410113000	7.090225000
H	-2.970301000	5.137547000	6.901364000
C	-4.322131000	2.450982000	5.155883000
H	-4.596849000	2.298935000	4.108678000
H	-3.554415000	1.729758000	5.430937000
H	-5.209487000	2.276928000	5.770044000
C	-4.912269000	4.882059000	4.976846000
H	-5.790201000	4.752454000	5.613809000
H	-4.546451000	5.904854000	5.092579000
H	-5.212628000	4.734097000	3.936958000
